# Proteome-scale tagging and functional screening in mammalian cells by ORFtag

**DOI:** 10.1038/s41592-024-02339-x

**Published:** 2024-07-05

**Authors:** Filip Nemčko, Moritz Himmelsbach, Vincent Loubiere, Ramesh Yelagandula, Michaela Pagani, Nina Fasching, Julius Brennecke, Ulrich Elling, Alexander Stark, Stefan L. Ameres

**Affiliations:** 1 Research Institute of Molecular Pathology (IMP), Vienna BioCenter (VBC), Vienna, Austria; 2 Vienna BioCenter PhD Program, Doctoral School of the University of Vienna and Medical University of Vienna, Vienna, Austria; 3 Max Perutz Laboratories, Vienna BioCenter (VBC), Vienna, Austria; 4 Department of Biochemistry and Cell Biology, Max Perutz Labs, University of Vienna, Vienna, Austria; 5 Institute of Molecular Biotechnology (IMBA), Vienna BioCenter (VBC), Vienna, Austria; 6 Medical University of Vienna, Vienna BioCenter (VBC), Vienna, Austria; 7 Present Address: Laboratory of Epigenetics, Cell Fate and Disease, Centre for DNA Fingerprinting and Diagnostics, Hyderabad, India; 8 Present Address: QUANTRO Therapeutics GmbH, Vienna, Austria

**Keywords:** High-throughput screening, Genetic mapping, Molecular engineering, Synthetic biology

## Abstract

The systematic determination of protein function is a key goal of modern biology, but remains challenging with current approaches. Here we present ORFtag, a versatile, cost-effective and highly efficient method for the massively parallel tagging and functional interrogation of proteins at the proteome scale. ORFtag uses retroviral vectors bearing a promoter, peptide tag and splice donor to generate fusions between the tag and endogenous open reading frames (ORFs). We demonstrate the utility of ORFtag through functional screens for transcriptional activators, repressors and posttranscriptional regulators in mouse embryonic stem cells. Each screen recovers known and identifies new regulators, including long ORFs inaccessible by other methods. Among other hits, we find that Zfp574 is a highly selective transcriptional activator and that oncogenic fusions often function as transactivators.

## Abstract (web summary)

This work presents ORFtag that enables proteome-wide functional screens by tagging and overexpression of endogenously encoded proteins via randomly integrated cassettes.

## Main

Proteins are central to almost all cellular processes, but their biochemical diversity often hinders systematic studies of protein function. Genetic loss-of-function screens—such as CRISPR–Cas9 and CRISPRi screens—are powerful methods for identifying genes involved in specific cellular processes, but typically do not provide direct insight into protein function^1^. They are also often hampered by functional redundancy and the essentiality of many genes. Conversely, sufficiency-based assays allow the direct determination of protein function^2,3^. However, current systematic methods rely on the delivery and expression of open reading frame (ORF) libraries (ORFeomes)^4,5^, which are not only costly and difficult to maintain, but also tend to favor shorter ORFs (<5 kb) due to limitations in DNA synthesis, cloning, viral packaging and delivery into cells^2^. Engineering of native gene locations can overcome these limitations^6^ and recent CRISPR–Cas9 techniques for systematic gene tagging have scaled to as many as 1,300 genes^7–13^, but achieving genome-wide coverage remains challenging. Here we present ORFtag, a versatile approach that allows for the massive, parallel and proteome-scale tagging and overexpression of endogenous genomically encoded ORFs.

## Results

### ORFtag overview

**Fig. 1:**
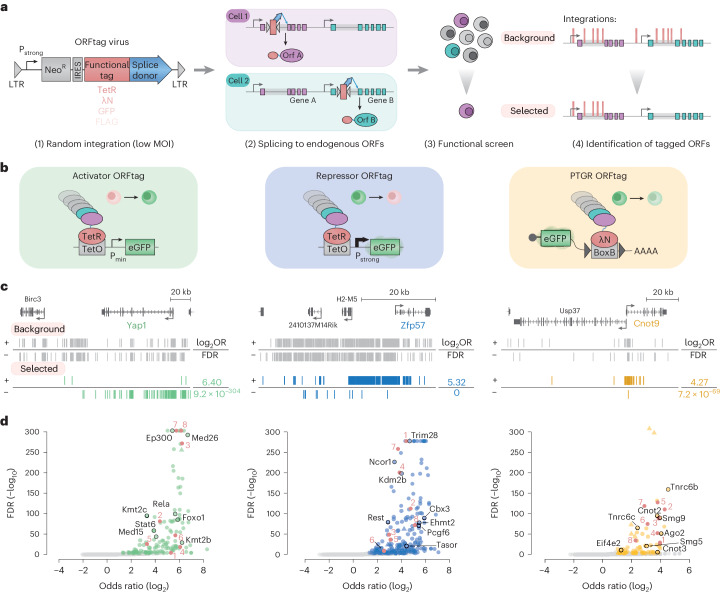
ORFtag is a versatile tool for proteome-wide functional assays. **a**, Overview of the ORFtag approach. MOI, multiplicity of infection; P_strong_, strong promoter. **b**, Schematic view of three different screens for transcriptional activators (green), repressors (blue) or PTGRs (yellow). eGFP, enhance GFP; P_min_, minimal promoter. **c**, Genome browser screenshots of ORFtag integration sites (vertical lines) in positive (+; top) or negative (–; bottom) strand direction, before (background; gray) and after FACS selection at the genomic locus of each activator (Yap1; green), repressor (Zfp57; blue) and PTGR (Cnot9; yellow) hit emerging from ORFtag screens in mES cells. The log_2_ odds ratio (log_2_OR) and FDR are indicated. **d**, Volcano plots highlighting known (black circles, names) and validated (marked red; Fig. 2d) hits for the three screens. Triangles indicate hits with unusual insertion patterns (Methods). Hits were identified using a one-tailed Fisher’s exact test on merged replicates. *P* values were corrected using the FDR method, with hits defined by FDR < 0.001 and log_2_ odds ratio ≥1.

**Extended Data Fig. 1:**
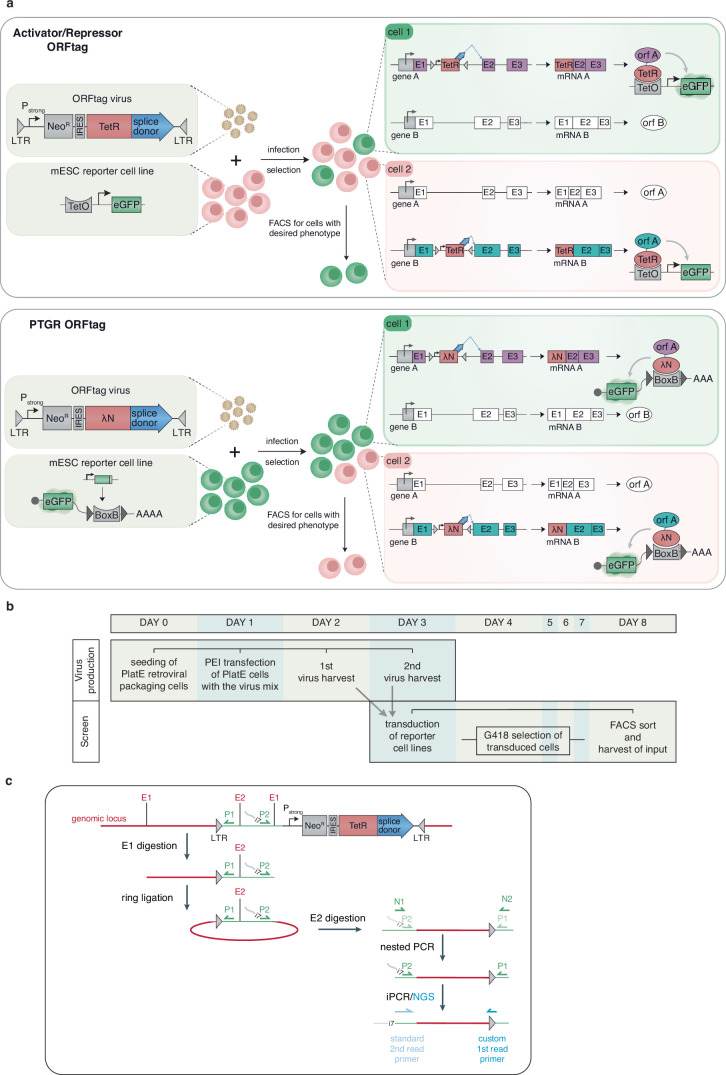
Overview of the ORFtag screening method. **a**, Detailed schematic illustrating Activator and Repressor ORFtag screens (top) and PTGR ORFtag screen (bottom). **b**, Graphical depiction of the ORFtag screening protocol. **c**, Visual representation of the inverse PCR followed by next-generation sequencing (iPCR-NGS) protocol.

**Extended Data Fig. 2:**
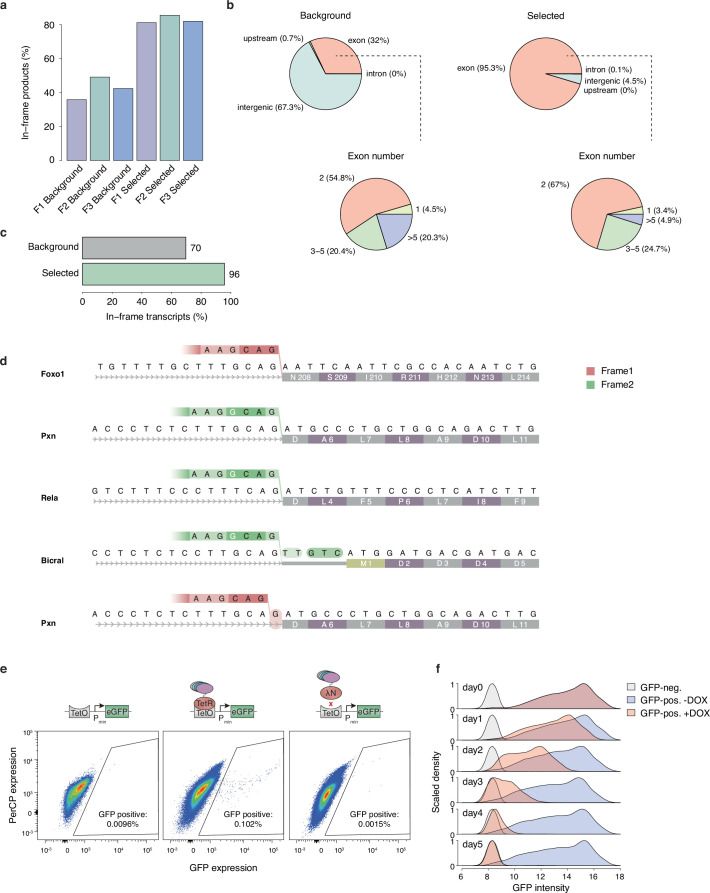
Validation of the ORFtag approach. **a**, Percentage of in-frame products for both background and selected samples across three reading frames based on ORFtag Activator screens performed with each of the three viruses separately. **b**, Distribution of sequences right downstream of the ORFtag cassette for background (left) and selected samples (right) based on ORFtag-targeted RNA-seq. Identity of spliced exons is shown below. **c**, Proportion of in-frame products for exon-spliced products from (b). **d**, Schematic of in-frame splicing events as determined by Sanger sequencing of five clones expressing ORFtag cassettes encoding Frame1 (red) or Frame2 (green). **e**, GFP expression and the proportion of GFP-positive cells are compared between the Activator reporter cell line alone and in cases of ORFtag screening with TetR (with recruitment) or λN (with no recruitment) functional tags. **f**, Density plots represent GFP levels in pre-sorted GFP positive cells, derived from an Activator ORFtag screen, over a 5-day period in the presence (red) or absence (blue) of Doxycycline. Parental non-activated reporter cell line is shown as control (grey).

ORFtag is based on insertional elements such as retroviral vectors containing a constitutively active promoter, a selection gene and a functional tag of interest followed by a splice donor sequence (Fig. 1a and Extended Data Fig. 1). Upon large-scale transduction of cultured cells, ORFtag cassettes randomly integrate into the genome and drive the transcription of nearby endogenous gene loci by splicing of the functional tag to splice-acceptor sites downstream of the integration site, creating near N-terminal fusion proteins. Since splice-acceptor sites within protein-coding exons can be in any one of three ORFs, a 1:1:1 mix of cassettes, one for each of the three ORFs, is used to perform pooled screening (see Extended Data Fig. 2a–d for an analysis and validation of this approach). ORFtag can be used to generate fusions of endogenous ORFs with a wide range of functional tags and it is compatible with diverse functional readouts including reporter-based positive selection by fluorescence-activated cell sorting (FACS). In the selected cell population, tagged genes are then identified by mapping integration sites using inverse PCR (iPCR) followed by next-generation sequencing (NGS) and by assessing the enrichment of insertions for each gene between selected and background samples^14^.

### Three functional screens benchmark ORFtag

To benchmark the ORFtag method, we performed a functional screen for transcriptional activators in mouse embryonic stem (mES) cells in two biological replicates (Fig. 1b and Extended Data Fig. 1). We systematically fused proteins to the DNA-binding domain of the bacterial tetracycline repressor (TetR), enabling their recruitdment to TetO binding sites located upstream of an integrated green fluorescent protein (GFP) reporter containing an inactive minimal promoter. To ensure that each cell expressed only one tagged ORF, we transduced reporter cells with retroviruses carrying ORFtag cassettes at low multiplicity of infection followed by selection. Cells with increased GFP expression, which control experiments attributed to the recruitment of candidates to TetO binding sites (Extended Data Fig. 2e,f), were isolated by FACS and insertion sites in the pool were determined by iPCR-NGS (Fig. 1a and Extended Data Fig. 1). Finally, we identified genes at which insertions were statistically over-represented in the sorted samples (‘selected’) compared to the nonselected background dataset (‘background’) by assigning each integration to the nearest downstream splice acceptor-containing exon of genomically encoded protein-coding genes (Fig. 1a, Methods and Supplementary Table 1).

We also performed a screen for transcriptional repressors, for which the GFP reporter was constitutively active and cells with reduced GFP expression were isolated by FACS (Fig. 1b and Extended Data Fig. 1), and a screen for posttranscriptional gene regulatory (PTGR) proteins (both screens in two replicates). For the latter, candidate ORFs were tagged with the lambda phage N (λN) protein to recruit them to boxB sites in the 3′ untranslated region (UTR) of a constitutively expressed GFP-encoding reporter messenger RNA (mRNA). Cells with reduced GFP expression indicated that tagged proteins repress GFP expression at the posttranscriptional level (Fig. 1b and Extended Data Fig. 1).

**Extended Data Fig. 3:**
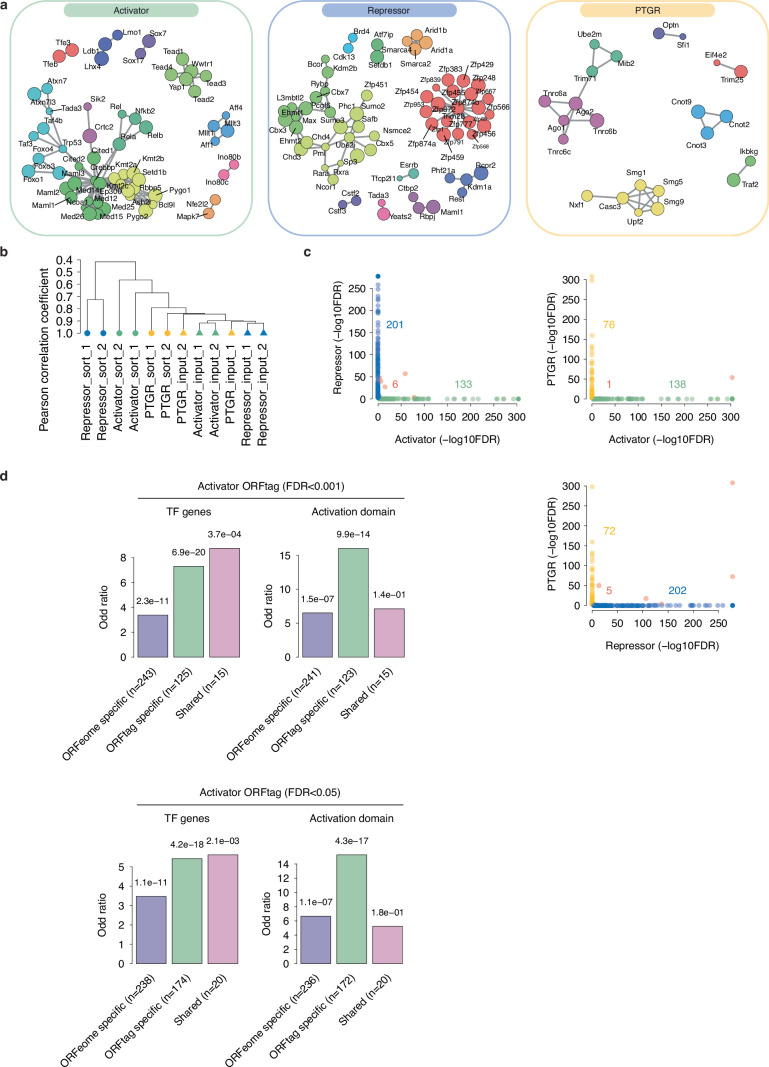
Analysis of ORFtag hits. **a**, STRING protein-protein interaction networks between activator/ repressor/ PTGR hits. Node communities were highlighted using a color code (Louvain method), and their size is proportional to the Odd ratio of the corresponding hit. Only the hits showing at least one interaction with another hit are shown. **b**, Dendrogram of Pearson Correlation Coefficients between background (input, triangles) and selected (sort, round) replicates from each functional screen. All background samples show high PCC ( ≥ 0.84). **c**, Scatter plots displaying a pairwise comparison of -log10(FDR) values for screened genes across different assays. **d**, Enrichment of Activator ORFtag screen hits and ORFeome screen hits for human homologous genes with annotated DNA-binding or activation domains. Shown are enrichments for hits called with the stringent (left) and lenient (right) FDR cutoff in the ORFtag screen. ORFtag hits exhibit two- to three-fold higher enrichment compared to ORFeome hits.

For each of the three screens, we found a prominent, screen-specific enrichment of insertions at positive control genes, exemplified by the transcriptional coactivator Yap1 (for the activator screen), the KRAB domain-containing Zfp57 (repressor) and the mRNA deadenylase complex subunit Cnot9 (PTGR) (Fig. 1c). In total, we identified 139 putative transcriptional activators, 207 repressors and 77 PTGR proteins using stringent thresholds (false discovery rate (FDR) <0.1%, log_2_ odds ratio ≥1; Supplementary Table 1 provides enrichment and FDR values for all genes, allowing analyses with relaxed cutoffs). Activator hits include several known transcriptional activators, such as p65, Ep300, Mediator complex subunits and all Kmt2(a-d) histone methyltransferases, which could not be screened previously due to their long ORFs of up to 17 kb (Fig. 1d and Extended Data Fig. 3a). Repressor hits include 75 KRAB zinc-finger repressors and their corepressor Trim28, HP1 family proteins, H3K9 methyltransferases and Polycomb repressive complex components (Fig. 1d and Extended Data Fig. 3a). Finally, the PTGR screen identified core components of the microRNA (Ago2, Tnrc6a/b/c) and nonsense-mediated decay (Smg1, Smg9, Upf2) pathways, members of the Ccr4-Not deadenylation complex (Cnot2, Cnot3, Cnot9) and translation inhibitors (Eif4e2, Eif4enif1) (Fig. 1d and Extended Data Fig. 3a).

**Fig. 2:**
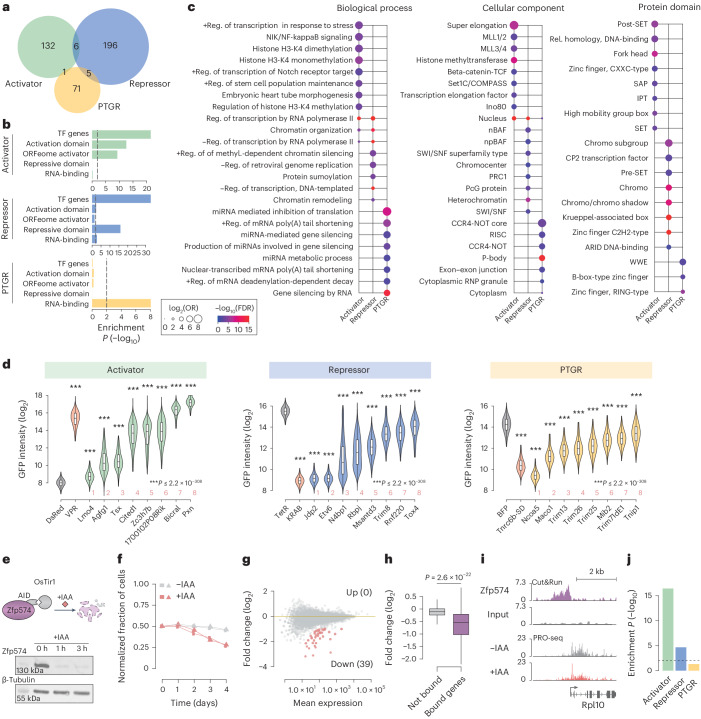
ORFtag interrogates protein function with high specificity. **a**, Overlap between activator (green), repressor (blue) and PTGR (yellow) hits. **b**, Enrichment of screen hits for human homologous genes with annotated DNA binding, activation, repressive domains and RNA-binding proteins. Additionally, activator screen hits are enriched for the ORFeome activator hits, although with only a limited overlap (*n* = 15; Extended Data Fig. 3d). Enrichment was assessed using a one-tailed Fisher’s exact test (alternative = ‘greater’), with intronic protein-coding genes as the background. TF, transcription factor. **c**, Top enriched protein domains, biological process and cellular component GO terms for activator, repressor and PTGR hits. Enrichment was assessed using a one-tailed Fisher’s exact test (alternative = ‘greater’), with intronic protein-coding genes as the background. *P* values were adjusted for multiple testing using the FDR method. miRNA, microRNA; reg., regulation. **d**, Independent validation of select screen hits. GFP intensity measured by flow cytometry in reporter cell lines stably expressing the indicated full-length proteins fused to TetR (activator, repressor) or λN (PTGR); one-sided Wilcoxon test (alternative = ‘greater’ for activator, and ‘less’ for repressor/PTGR hits), ***P ≤ 2.2 × 10^−308^. The sample size was 25,000 cells for each validation, except for N4bp1 (*n* = 5,766) and Trim8 (*n* = 3,775). Refer to Fig. 1d for the position of the hits in the volcano plot. Box plots show the median (line), upper and lower quartiles (box) ± 1.5 × interquartile range (whiskers); outliers are not shown. **e**, Schematic view of Zfp574 rapid depletion using AID. Western blot analysis demonstrates the rapid depletion of Zfp574 following treatment with IAA. This result was consistently observed in two independent experiments. **f**, Cell viability timecourse in the presence (−IAA, in gray) or absence of Zfp574 (+IAA, in red). Shown are two biological replicates. **g**, MA plot showing PRO-seq fold changes (log_2_) after 6 h depletion of Zfp574. Significantly up- (0) or downregulated (39) genes are highlighted in red. **h**, PRO-seq fold changes (log_2_) of not-bound (*n* = 12,381) versus Zfp574 promoter-bound genes (*n* = 105) after Zfp574 depletion; two-sided Wilcoxon test. Box plots show the median (line), upper and lower quartiles (box) ± 1.5 × interquartile range (whiskers); outliers are not shown. **i**, Zfp574 Cut&Run and PRO-seq screenshots at the Rpl10 locus. **j**, Enrichment of screen hits for genes that were identified as part of oncogenic fusions. Source data

While ORFtag integrations were highly reproducible for each screen (Extended Data Fig. 3b), the hits from the three different biological assays showed almost no overlap, indicating that ORFtag does not lead to the recurrent and artifactual detection of nonspecific genes (Fig. 2a and Extended Data Fig. 3c). Consistent with this, the activator and repressor screen hits were highly enriched for proteins containing activating or repressive domains, respectively, and both protein sets share a significant enrichment for known transcription factors. Moreover, the activator screen hits were specifically enriched for proteins identified by the ORFeome screen for transcriptional activators^2^ (Fig. 2b and Extended Data Fig. 3d), and only PTGR hits were enriched for known RNA-binding proteins (Fig. 2b). In addition, the genes identified by the three screens were enriched for different gene ontology (GO) terms and protein domains, all of which are consistent with their associated functions (Fig. 2c). Taken together, these results indicate that ORFtag is compatible with diverse functional assays and delivers assay-specific hits.

### ORFtag recovers known and identifies novel regulators

To experimentally validate the screen results at the level of protein-inherent functionality, we selected eight hits from each screen across a wide range of enrichment scores, focusing on hits for which a direct function in transcriptional activation or repression or in PTGR had not been demonstrated. We individually cloned and transduced each hit fused to the respective TetR or λN tags, and tested whether this was sufficient to regulate the respective reporters. All candidates tested, including hits that were not previously linked to the respective biological processes, could be validated in recruitment assays together with previously known regulators, confirming that ORFtag screens are highly specific and have low false positive rates (Fig. 2d). For example, recruitment of the annotated cytoskeletal protein Pxn or the uncharacterized protein 1700102P08Rik was sufficient to strongly activate transcription, whereas the E3 ubiquitin ligase Trim8 and the uncharacterized protein Msantd3 were sufficient to repress transcription. By contrast, the neuronal activity-associated protein Maco1 and the E3 ubiquitin ligase Trim13 were sufficient to repress reporter gene expression when recruited to the 3′ UTR of an mRNA.

To assess the potential of ORFtag in assigning cellular roles to uncharacterized proteins, we sought to investigate the endogenous function of the zinc-finger protein Zfp574, which ORFtag specifically identified as a transcriptional activator. Using the auxin-inducible degron (AID) system (Fig. 2e), we showed that depletion of Zfp574 results in a notable growth defect (Fig. 2f), indicating that Zfp574 is essential for cellular fitness. Rapid depletion of Zfp574 followed by PRO-seq further revealed that Zfp574 functions strictly as a transcriptional activator, consistent with the ORFtag results (39 genes go down, and 0 genes go up after depletion of Zfp574 at FDR ≤ 0.05 and a fold change ≥2) (Fig. 2g). Cut&Run for Zfp574 identified 140 binding sites genome-wide, most (87.9%) of which are located in promoter-proximal positions (±500 bp around the gene transcription start sites (TSSs)), and transcription of the promoter-bound genes was strongly affected after Zfp574 depletion (Fig. 2h,i). Thus, Zfp574 is a selective transcriptional activator that specifically binds and activates a small set of genes that support cell fitness. Taken together, our results demonstrate that ORFtag, coupled with functional assays, provides a robust and powerful method for the high-throughput assignment of protein function.

### ORFtag assays biochemical function rather than cellular role

Some identified hits may regulate gene expression in ORFtag assays without necessarily doing so endogenously. This underscores the distinction between the inherent biochemical function of a protein (as evaluated here) and the protein’s role within the cell^1^. In fact, the process of tagging and/or chromatin- or RNA-tethering alters a protein’s expression level and can overwrite a protein’s usual cellular function and localization within the cell (for example N-terminal signaling peptides can be bypassed, replaced or overwritten by ORFtag). These hits are valuable because their ability to activate and/or repress gene expression in principle is highly relevant, for example in cancer, when chromosomal rearrangements create oncogenic fusion proteins. Indeed, among our hits is the ortholog of the oncogene *C3orf62*, recently described by a tethering-based approach to be an activator^2^. We systematically compared the ORFtag hits with their human orthologs and found that oncogenes are enriched among activators and, to a lesser extent, repressors but not among posttranscriptional regulators (Fig. 2j). These include, for example, *Zc3h7b* and *D630045J12Rik* (*KIAA1549* in human), which can function as activators, and *Gm10324* that can function as a repressor, highlighting that oncogenic fusions can recruit unrelated genes to function in gene regulation.

### ORFtag leads to near-N-terminal tagging of most genes

**Fig. 3:**
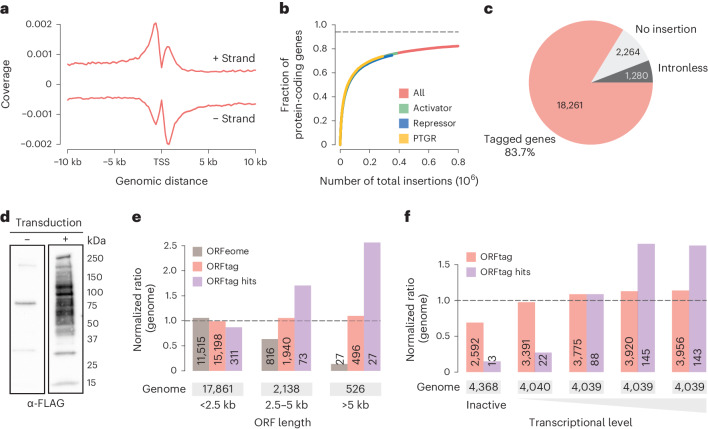
Scope and limitations of massive parallel protein tagging using ORFtag. **a**, Distribution of ORFtag integrations around TSSs of mouse protein-coding genes. **b**, Saturation curve displaying the relationship between the fraction of tagged proteins and the number of determined integration sites. **c**, Fraction of genes showing at least one integration in the combined background sample. **d**, Western blot against the FLAG tag assessing the tagging pattern in mES cell lysate before (−) and after (+) ORFtag transduction. This analysis was conducted on the first replicate of the PTGR screen. **e**, Ratio of protein-coding genes that were successfully tagged using ORFtag (ORFtag; pink) or were hits in any of the three screens (ORFtag hits; purple), compared to the distribution of ORF lengths across the whole mouse genome (genome; dashed line). Human ORFeome is shown for comparison (ORFeome; light gray). See Methods for further details. **f**, Ratio of protein-coding genes that were successfully tagged using ORFtag (ORFtag; pink) or were hits in any of the three screens (ORFtag hits; purple), compared to the distribution of ORF expression levels across the whole mouse genome (genome; dashed line). Source data

**Extended Data Fig. 4:**
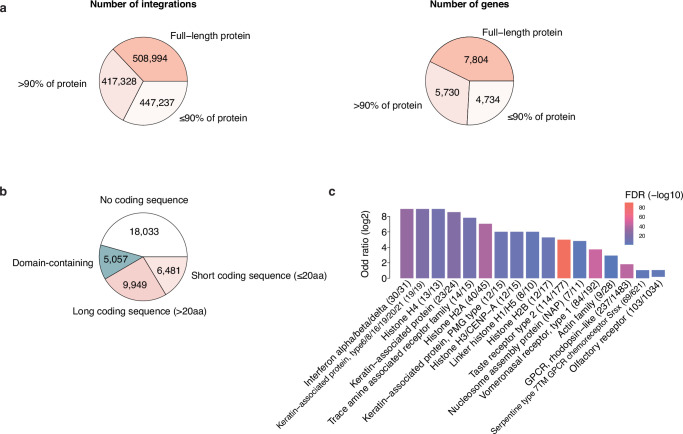
Global assessment of ORFtag integrations. **a**, Fraction of integrations (left) and genes (right) wherein splicing results in capturing either full-length proteins, over 90% of the coding sequence, or less. **b**, Fraction of exons that are non-coding (in white), code either for no known protein domain (in shades of pink) or for a high confidence annotated protein domains in the Pfam database (Domain-containing CDS, in blue). Importantly, most first exons are non-coding or do not contain specific protein domains, fostering the use of ORFtag for a wide range of functional studies. **c**, Protein family enrichment of intronless genes, whose majority belongs to few protein families. Enrichment was assessed using a one-tailed Fisher’s exact test (alternative= “greater”), employing the total number of protein-coding genes as the background. P-values were adjusted for multiple comparisons using the FDR method.

Having established that retroviral integration sites represent indeed successful ORF tagging events that score in functional assays, we undertook a systematic and critical evaluation of ORFtag’s ability to comprehensively and reproducibly tag proteins. Comparison of the retroviral integration sites from six independent transductions, performed in three different laboratories, revealed that, regardless of the protein tag used, experimental cassettes integrated in a similar distribution across the genome and the number of insertions per genomic region were highly correlated (Pearson correlation coefficient (PCC) ≥ 0.84) (Extended Data Fig. 3b). ORFtag integrations were enriched near TSS, a well-known feature of retroviral vectors^14^, allowing the tagging of near full-length proteins (Fig. 3a and Extended Data Fig. 4a). Assigning each integration to a gene locus revealed that, given the scale and sequencing depth of our screens, we were able to tag at least 83.7% of all mouse protein-coding genes with a median count of 15 integrations per gene (Fig. 3b,c). The tagged genes include those with large ORFs yielding high molecular weight proteins (Fig. 3d). Indeed, in contrast to ORFeome-based approaches that are biased toward short ORFs, ORFtag is not affected by gene length (Fig. 3e). Furthermore, the retroviral ORFtag cassette allowed the tagging of ORFs with different endogenous expression levels, including more than 59% of genes that are not normally expressed in mES cells (Fig. 3f), and allows all tested candidates to be expressed at similar levels. The hits identified in the three functional screens also include genes of different length and expression levels (Fig. 3e,f).

A limitation of ORFtag lies in its inability to functionally probe intronless genes and first exons, due to the lack of splice-acceptor sites. However, it is worth noting that 45.6% of first exons are noncoding and that among protein-coding first exons, the median length of the encoded peptide is 31 amino acids short. As a result, only 12.8% of first exons contain annotated protein domains (Extended Data Fig. 4b). Intronless genes, which cannot be tagged, represent only a small fraction of protein-coding genes (5.9%). These are dominated by a few protein families, including histones and various sensory receptors (Extended Data Fig. 4c), leaving more than 90% of protein-coding genes as potentially taggable by ORFtag. We also note that certain genes may not be accessible to ORFtag screens if cellular fitness is sensitive to changes in their expression levels.

## Discussion

In summary, ORFtag is an easy-to-implement functional genomics tool that enables cost-effective proteome-scale functional screens, providing an alternative to ORFeome-based approaches with broader gene coverage, especially for long ORFs. Based on tagging and overexpressing endogenous genomically encoded proteins, it is ideally suited to investigate the proteins’ inherent biochemical functions in ‘cellular biochemistry’-like assays, as opposed to ‘cellular physiology’-like assays that study the proteins’ cellular roles. ORFtag can incorporate diverse functional tags for a wide range of screens, such as intracellular protein localization using fluorescence markers, confining proteins to specific compartments via signal peptides or facilitating proximity-induced dimerization domains for studying proteins involved in signaling, degradation or stabilization. Furthermore, ORFtag could serve as an alternative to CRISPRa by activating endogenous genes with ORFtag cassettes that contain small affinity tags or even just a 5′ UTR and start codon. Finally, ORFtag can be readily used in cellular systems of various model organisms without the need to generate species-specific resources. This adaptability and versatility make ORFtag a promising tool for advancing functional genomics research.

## Methods

### Cell culture conditions

All experiments presented here were carried out in diploid mES cells that were derived from originally haploid HMSc2 termed AN3-12 (ref. ^14^) and were obtained from the Institute of Molecular Biotechnology (IMBA) Haplobank. The mES cells were cultivated without feeders in high-glucose-DMEM (Sigma-Aldrich) supplemented with 13.5% fetal bovine serum (Sigma-Aldrich), 2 mM l-glutamine (Sigma-Aldrich), 1× penicillin-streptomycin (Sigma-Aldrich), 1× MEM nonessential amino acid solution (Gibco), 1 mM sodium pyruvate (Sigma-Aldrich), 50 mM β-mercaptoethanol (Merck) and in-house produced recombinant leukemia inhibitory factor. Virus packaging cell lines, Lenti-X 293T (Takara) and PlatinumE (Cell Biolabs), were grown according to the manufacturer’s instructions. *Drosophila* S2 cells (obtained from Thermo Fisher, cat. no. R69007) were maintained in Schneider’s *Drosophila* Medium supplemented with 10% heat‐inactivated fetal bovine serum (Sigma-Aldrich). All mammalian cell lines were cultured at 37 °C and 5% CO_2_, S2 cells were cultured at 27 °C and 0.4% CO_2_. All cell lines were regularly tested for mycoplasma contamination.

### Reporter cell lines

The reporter cell line for the ‘repressor’ screen was established previously^15^ and contains the reporter construct inserted into the expression-stable locus on Chr15 that is compatible with the Flp recombinase-mediated cassette exchange (RMCE). The reporter cell line for the ‘activator’ screen was generated by RMCE as follows: 5 × 10^6^ cells were electroporated with a mix of 10 µg of plasmid containing constructs flanked by FRT/F3 sites, and 6 µg of plasmid expressing Flp, using a Maxcyte STX electroporation device (GOC-1) and the Opt5 program. Seven days after the transfection, cells were sorted and clonal cell lines were generated. Cell lines were genotyped using integration-site-specific PCRs and Sanger sequencing. The activator reporter construct contains the PuroR-IRES-GFP reporter under the control of the minimal promoter derived from the *MYLPF* gene (chr16:30374730–30374857+, hg38) that was shown to have a low basal expression and high inducibility^16^. Upstream of the promoter are 7× TetO sites flanked by the loxP sites.

The reporter cell line for the PTGR screen was created by nucleofection of AN3-12 mES cells with 500 ng of the reporter construct and 10 µg of a Tol2 transposase encoding plasmid using the Mouse ES Cell Nucleofector Kit (Lonza) according to the manufacturer’s protocol using an Amaxa Nucleofector (Lonza). The PTGR reporter construct encodes for PuroR-IRES-GFP followed by ten boxB sites that are flanked by two loxP sites under the control of a PGK promoter. Cells were subsequently selected using 1 µg ml^−1^ Puromycin (Gibco) followed by single clone selection. Single cell clones were afterward transduced with a retroviral vector for the expression of pMSCV_hygro_CreERT2 and selected with 250 µg ml^−1^ Hygromycin (Roche) followed by single cell clone selection.

### ORFtag screens

The ORFtag viral constructs were derived from the ecotropic Retro-EGT construct^14^ that includes the sequence features necessary for the iPCR protocol (detailed below and in Extended Data Fig. 1). Furthermore, the construct features a constitutively active PGK promoter that drives the expression of a *NeoR* resistance gene separated from a tag by the internal ribosome entry site (IRES) sequence. The tag contained either TetR with an N-terminally located nuclear localization signal (activator screen, repressor screen; Addgene IDs 22098, 220982, 220983) or LambdaN domain (PTGR screen; Addgene IDs 220984, 220985, 220986). Additionally, the tag includes a 2× GGGS-linker followed by the BC2-tag and 3xFLAG-tag. Finally, the ORFtag construct contains a consensus splice donor motif (GT) followed by a segment of the Hprt intron (chrX:53020400–53020556+, mm10). To ensure tagging of genes in all three possible coding frames, three variants of the constructs were used that contain 0, 1 or 2 additional nucleotides upstream of the consensus splice motif (GT), resulting in the following sequence: AAG-CAG-GT (frame 1), AAG-**G**-CAG-GT (frame 2) or AAG-**GC**-CAG-GT (frame 3) where AAG represents the last codon of the 3xFLAG-tag.

Apart from the ORFtag test depicted in Extended Data Fig. 2, where each of the three ORFtag constructs were used separately, we used a balanced mixture (1:1:1) of the three ORFtag plasmids before transfection. The ORFtag retroviral constructs were packed in PlatinumE cell lines using polyethylenimine (PEI) reagent as described previously^14^. Specifically, PlatinumE cells were seeded 24 h before transfection (11.25 million cells per 150 mm cell culture dish and no antibiotics). A mixture consisting of 45 µg of ORFtag plasmid mix, 15 µg of pCMV-Gag-Pol plasmid (Cell Biolabs), 135 µl of PEI and high-glucose-DMEM medium without supplements was prepared to a total volume of 3.2 ml per 15 cm cell culture dish. After a 20 min incubation period, the mixture was gently added to the cell culture. After 12 h, the medium was replaced with the fresh mES cell medium, and the virus was harvested twice after 12 and 24 h and pooled.

Reporter cell lines (100–150 million cells) were seeded 4 h before transduction in 245 × 245 mm^2^ square dishes and transduced with packaged retrovirus in the presence of 6 µg ml^−1^ polybrene (Sigma) to ensure low transduction efficiency (<20%) and thus the introduction of only one virus per cell (as detailed later). Cells were gathered 24 h later and plated in medium containing 0.1 mg ml^−1^ G418 (Gibco) for selection of transduced cells. Selection was continued until all cells on the control plate died (no transduction, 4–5 days), with two washes with 1× PBS and medium exchange every day. After selection, 40 million cells were processed as nonselected background for mapping of genomic integrations (below). The remaining cells were sorted for GFP-positive (activator screen) or GFP-negative (repressor screen) populations using BD FACSAria III or IIu cell sorters (BD Biosciences) and processed for mapping of genomic integrations (below). Refer to Supplementary Fig. 1 exemplifying the gating strategy.

For the PTGR screen, a five-sort strategy was applied to enrich cells that show a tethering dependent repression of reporter gene expression. Cells with a GFP expression equal to the lowest 10% of GFP expression observed after selection were sorted using BD FACSAria III and expanded thereafter. Additionally, nonsorted cells were maintained for gating of the consecutive sorts. Two additional sorts for cells with GFP expression similar to the lowest 10% of GFP signal observed in the nonsorted cells were performed and again expanded in between the sorts. A fourth sort was performed for cells with a GFP expression equal to the lowest 5% of GFP signal observed in the nonsorted cells. After expansion, the cells were treated with 500 nM 4-hydroxytamoxifen (Sigma) to induce Cre-mediated recombination and to flox the boxB sites of the reporter construct and hence to revert the tethering. Thereafter a final sort was performed to select a cell population with a GFP expression equal to the highest 70% of GFP expressing cells.

Transduction efficiency was measured with every ORFtag screen by plating 10,000 cells on a 150 mm dish and selecting with G418 (Gibco). A control plate with 1,000 cells was also plated without selection. After 10 days, colonies were counted and transduction efficiency was calculated as the number of colonies on the selected plate divided by the total number of cells plated (ten times the number of colonies on the control plate). The following transduction rates were determined: activator screen (6.1 and 7.6%), repressor screen (7.5 and 6.9%) and PTGR screen (16 and 34%). Using these rates, the probability of multi-transduction events was calculated through a Poisson distribution: activator screen (0.18 and 0.27%), repressor screen (0.27 and 0.23%) and PTGR screen (1.15 and 4.62%).

### Mapping of genomic integrations by NGS

Genomic locations of ORFtag integrations were mapped using a modified iPCR-NGS protocol^14^ (refer to Extended Data Fig. 1c for a detailed schematic). Genomic DNA was prepared by lysing cell pellets in lysis buffer (10 mM Tris-HCl pH 8.0, 5 mM EDTA, 100 mM NaCl, 1% SDS, 0.5 mg ml^−1^ proteinase K) overnight at 55 °C. After cell lysis, RNA A treatment (Qiagen, 100 mg ml^−1^, 1:1,000 dilution) was performed for 2 h 37 °C, followed by a series of two extractions using phenol:chloroform:isoamyl alcohol and one extraction using chloroform:isoamyl alcohol. The samples then underwent two separate digestion reactions (with up to 4 µg of genomic DNA) using NlaIII and MseI enzymes (NEB) overnight at 37 °C, followed by purification using a Monarch PCR&DNA Cleanup Kit (NEB). Ring ligation was carried out using T4 DNA ligase (NEB) overnight at 16 °C, followed by heat-inactivation (65 °C, 15 min) and linearization using SbfI-HF (NEB) for 2 h at 37 °C. The digested DNA was then purified using a Monarch PCR&DNA Cleanup Kit (NEB) and amplified using first a nested PCR reaction with KAPA HiFi HotStart ReadyMix (Roche), and a specific primer pair (TGCAGGACCGGACGTGACTGGAGTTC*A, TGCAGGACGATGAGCAGAGCCAGAACC*A) for 16 cycles. After clean-up with AMPure XP Reagent (Beckman Coulter, 1:1 ratio beads:PCR), iPCR amplification was carried out with KAPA HiFi HotStart ReadyMix (Roche) and a specific primer pair (AATGATACGGCGACCACCGAGATCTACACGAGCCAGAACCAGAAGGAACTTGA*C, CAAGCAGAAGACGGCATACGAGAT (custom-barcode) GTGACTGGAGTTCAGACGTGTGCTCTTCCGATCT) for 18 cycles. Afterward, amplified libraries were size selected for a range of 400–800 bp using SPRIselect beads (Beckman Coulter). NGS was performed on an Illumina NextSeq550 or llumina HiSeq 2500 sequencer according to the manufacturers’ protocols with custom first-read primer (1:1 mix of GAGTGATTGACTACCCGTCAGCGGGGGTCTTTCA and TGAGTGATTGACTACCCACGACGGGGGTCTTTCA). Refer to Supplementary Fig. 2 for a complete list of primers.

### ORFtag RNA-seq

Expanded GFP-positive cells and background cells (40 million each) from activator ORFtag screen were collected, and total RNA extraction was carried out using the RNeasy Maxi kit (Qiagen, cat. no. 75162) with β-mercaptoethanol supplemented RLT buffer. Subsequently, mRNA was isolated using Oligo-dT25 beads (Invitrogen, cat. no. 61005), followed by TurboDNase I treatment (Invitrogen, cat. no. AM2238) at 37 °C for 30 min. The purified mRNA was cleaned with AMPure XP beads (Beckman Coulter, cat. no. A63882) at a ratio of 1:1.8 (RNA:beads), followed by reverse transcription using SuperScript III (Invitrogen, cat. no. 8080093) with a random hexamer primer at the final concentration 0.2 µM (GTGACTGGAGTTCAGACGTGTGCTCTTCCGATCTNNNNNN) and the following conditions: 25 °C for 5 min, 50 °C for 1 h and 70 °C for 15 min. Afterward, complementary DNA (cDNA) was treated with RNaseA (Thermo Fisher, cat. no. EN0531) at 37 °C for 1 h, followed by clean-up using AMPure XP beads at a ratio of 1:1.4. Library amplification was performed using a seminested PCR approach. Initially, 5 µl of cDNA was amplified in ten PCR reactions using KAPA HiFi HotStart ReadyMix (Roche) and the following primers (forward: TATGTGGCCTGGAGAAACAGCTA and reverse: GTGACTGGAGTTCAGACGTGTGCTCTTCCGATCT) under the following conditions: 98 °C for 45 s; 12 cycles of 98 °C for 15 s, 65 °C for 30 s, 72 °C for 30 s; followed by 72 °C for 60 s. The first amplification was followed by clean-up using AMPure XP beads at a ratio of 1:1.4. Subsequently, a second PCR in ten reactions was performed using KAPA HiFi HotStart ReadyMix and the following primers (forward: CACGACGCTCTTCCGATCTNNNNNNCCACGACGGAGACTACAAGG and reverse: GTGACTGGAGTTCAGACGTGTGCTCTTCCGATCT) under the following conditions: 98 °C for 45 s; 5 cycles of 98 °C for 15 s, 65 °C for 30 s, 72 °C for 30 s; followed by 72 °C for 60 s. This was followed by a clean-up using AMPure XP beads at a ratio of 1:1.4. The final library amplification was carried out using the KAPA HiFi HotStart Real-Time Library Amp Kit (Roche) with Illumina Truseq Small RNA library amplification kit primers (eight cycles of amplification). Finally, the amplified libraries were size selected for a range of 200–800 bp using SPRIselect beads (Beckman Coulter). Refer to Supplementary Fig. 2 for a complete list of primers.

### ORFtag cassette splicing in GFP-positive cells

Expanded clones of GFP-positive cells from activator ORFtag screen were collected, and total RNA extraction was carried out using the RNeasy Mini kit (Qiagen, cat. no. 74104) with β-mercaptoethanol supplemented RLT buffer, followed directly by TurboDNase I treatment (Invitrogen, cat no. AM2238) at 37 °C for 30 min. The purified RNA was cleaned with AMPure XP beads (Beckman Coulter, cat no. A63882) at a ratio of 1:1.8 (RNA:beads), followed by reverse transcription using SuperScript III (Invitrogen, cat. no. 18080093) with a random hexamer primer at the final concentration 0.2 µM (GTGACTGGAGTTCAGACGTGTGCTCTTCCGATCTNNNNNN) and the following conditions: 25 °C for 5 min, 50 °C for 1 h and 70 °C for 15 min. Afterward, cDNA was treated with RNaseA (Thermo Fisher, EN0531) at 37 °C for 1 h, followed by clean-up using AMPure XP beads at a ratio of 1:1.4. Amplification was performed using 5 µl of cDNA in a PCR reaction using KAPA HiFi HotStart ReadyMix (Roche) and the following primers (forward: CCTGGCAATCGAGATGCTGGACAG and reverse: GTGACTGGAGTTCAGACGTGTGCTCTTCCGATCT) under the following conditions: 98 °C for 45 s; 33 cycles of 98 °C for 15 s, 65 °C for 30 s, 72 °C for 30 s; followed by 72 °C for 60 s. The final product was run on a gel and the most prominent band was extracted and Sanger sequenced using CCTGGCAATCGAGATGCTGGACAG primer. Refer to Supplementary Fig. 2 for a complete list of primers.

### ORFtag dependency on candidate recruitment

To validate that GFP expression directly reflects candidate recruitment, the Tet-OFF system present in activator ORFtag screens was used. Expanded GFP-positive cells were cultured in the presence or absence of Doxycyclin (final concentration 1 µg ml^−1^). GFP expression was measured over a period of 5 days using LSR Fortessa (BD) flow cytometer. FlowJo (v.10.10) and R package flowCore (v.2.12.2) were used for processing and visualization.

### Immunoprecipitation

To confirm expression of tagged proteins, the PTGR reporter mES cells, transduced with the ORFtag construct as well as nontransduced cells, were lysed in lysis buffer (50 mM Tris-HCl pH 7.5, 150 mM NaCl, 0.1% SDS, 1% Triton-X-100, 0.5% NP-40, 0.5 mM EDTA) supplemented with Proteinase Inhibitor (Roche) and protein concentration was determined photometrically using the Protein Assay Dye Reagent Concentrate (BioRad), according to the manufacturer’s protocol and photometric measurement at 595 nm. Tagged proteins were captured using 80 µl of in-house produced BC2-nanobody coupled magnetic beads from 1 mg total protein. Bound proteins were eluted by resuspension of the beads in 1× SDS-sample buffer and incubated at 95˚C for 5 min. Further details about western blotting can be found below.

### Western blotting

For the Zfp574 experiments, 3 million cells were collected, centrifuged at 300*g* for 5 min, washed with 1× PBS and lysed in 100 µl RIPA buffer containing protease inhibitor (Roche) and Benzonase (Sigma-Aldrich). For complete lysis, cells were incubated on ice for 30 min and sonicated for 5 min (30 s on/off, Diagenode Bioruptor). Afterward, samples were centrifugated for 5 min at full speed and 4 °C, and supernatants were supplemented with 20 µl of 4× Laemmli buffer with 10% β-mercaptoethanol. Samples were boiled for 5 min at 98 °C.

Proteins were resolved on SDS–PAGE on a 4–15% Mini-PROTEAN TGX Precast Protein Gel (BioRad) and transferred to an Immobilon-P polyvinyl difluoride membrane (Merck Millipore) using a wet-chamber system. Tagged proteins were detected using mouse α-Flag M2 (Sigma-Aldrich, cat. no. F3165, 1:10,000), mouse α-V5-tag (Thermo Fisher R960-25, 1:1,000) or rabbit α-β-tubulin (Abcam, ab6046, 1:10,000) as primary and HRP-α-Mouse (Cell Signaling, cat. no. 7076, 1:10,000) or HRP-α-Rabbit (Cell Signaling, cat. no. 7074, 1:10,000) as secondary antibody and imaged using ClarityTM Western ECL Substrate (BioRad) with a ChemiDocTM Imaging System (BioRad) using ImageLab v.5.1.1 (BioRad).

### Individual recruitment validations

To validate activator hits, the candidates were amplified by PCR from mES cell cDNA and inserted into retroviral constructs that comprises the PGK promoter that drives the expression of a *PuroR* resistance gene and a tag separated by the IRES sequence. The tag contains TetR, along with an N-terminal nuclear localization signal, a 2× GGGS-linker, a BC2-tag and a 3xFLAG-tag, followed by the tested candidate. Refer to Supplementary Fig. 2 for a complete list of primers. Retroviral constructs were packed in PlatinumE cell lines (above), and the activator reporter cell line (170,000 cells) was transduced in the presence of 6 µg ml^−1^ Polybrene (Sigma). Cells were collected 24 h later and plated in medium containing 1 µg ml^−1^ Puromycin (InvivoGen) to select for transduced cells. After 5 days of selection, the reporter expression was analyzed on an LSR Fortessa (BD) flow cytometer. For processing and visualization, FlowJo (v.10.10) and R package flowCore (v.2.12.2) was used. Refer to Supplementary Fig. 1 exemplifying the gating strategy.

To validate repressor and PTGR hits, PCR was used to amplify the candidates from mES cell cDNA, and lentiviral plasmids were created as fusion proteins containing TetR/lamdaN-Candidate-P2A-mCherry coding sequence under the control of an EF1a promoter. For the validation of Trim71, cDNA excluding the fragment encoded in exon 1 (Trim71dE1) was cloned into the aforementioned lentiviral plasmid. A fragment encoding for the silencing domain of human Tnrc6b (Tnrc6b-SD) was expressed using the same lentiviral plasmid as above as a positive control for the validation of PTGR hits. Refer to Supplementary Fig. 2 for a complete list of primers. Lentivirus was produced in Lenti-X 293T cells as in ref. ^15^. Repressor and PTGR reporter cells were then transduced with the virus in the presence of 8 µg ml^−1^ Polybrene (Sigma). After 7 days of transduction, reporter expression was analyzed on an LSR Fortessa (BD) flow cytometer. Reporter cells transduced with recruitment constructs were gated based mCherry expression. For processing and visualization, FlowJo (v.10.10) and R package flowCore (v.2.12.2) was used.

### AID cell line generation

A parental cell line expressing the E3 ligase for the AID was generated by inserting a cassette into the expression-stable locus on Chr15 that is compatible with the Flp RMCE in mES cells (‘Reporter cell lines’ section). The construct contained EF1alpha- ARF16- HA- P2A- OsTir1- 3xMyc- T2A- mCherry- SV40_polA site flanked by the FRT/F3 sites. The clonal Tir1 parental cell line was genotyped using integration-site-specific PCRs and Sanger sequencing.

To generate the N-terminally AID-tagged Zfp574 cell line, 5 × 10^6^ Tir1 parental cells were transfected with 10 µg of plasmid^17^ that expresses Cas9 and the genomic RNA against a target locus (CTTGCTGCTGCCATGACTG) and 5 µg of plasmid with a knock-in cassette containing Blasticidin-P2A-V5-AID-GGGS flanked by 20 bp microhomology arms^17^ using a Maxcyte STX electroporation device (GOC-1) and the Opt5 program. Two days after the transfection, cells were selected for knock-ins with 10 µg ml^−1^ Blasticidin (Thermo Fisher), individual clones were genotyped using knock-in-site-specific PCRs and Sanger sequencing. Refer to Supplementary Fig. 2 for a complete list of primers. Potential candidates were investigated by western blotting against the integrated V5-tag (Thermo Fisher, cat. no. R960-25) with or without 500 μM 3-indoleacetic acid (IAA) (Merc) treatment.

### Cell viability timecourse

For growth curve assays, AID-tagged cell line (mCherry positive, section ‘AID cell line generation’) was mixed at a 1:1 ratio with mouse ES wild-type cells, split into control (−IAA) and treatment (+IAA, Merc, 500 μM) groups and cultured in a 24-well cell culture plate. The ratio between mCherry positive and negative cells was quantified every 24 h by flow cytometry (iQue Screener PLUS, Intellicyt).

### PRO-seq

For each condition, 1 × 10^7^ AID-Zfp574 cells were collected and nuclei were isolated after 6 h of 500 μM IAA treatment or no treatment (two biological replicates per condition). Spike-in control (S2 *Drosophila* cells; 1% of mES cells) were added at the level of nuclei permeabilization step. The next steps of the PRO-seq protocol were performed as in ref. ^18^ with a single modification: the nuclear run-on was performed at 37 °C for 3 min.

### Cut&Run

For each biological replicate, 1 × 10^6^ cells from the AID-Zfp574 cell line or the Tir1 parental cell line were used. The Tir1 parental cell line is used as input, each experiment was performed in two biological replicates. The protocol was performed as in ref. ^19^ with a V5-tag antibody (Thermo Fisher, cat. no. R960-25) that was added to a final dilution of 1:100.

### Bioinformatic analyses

All bioinformatic analyses were performed in R (v.4.2.0, https://www.R-project.org/). Computations on genomic coordinates were conducted using the GenomicRanges (v.1.50.1)^20^ and the data.table (v.1.14.6, https://CRAN.R-project.org/package=data.table) R packages. Genomic data were visualized using IGV browser (v.2.16.0). All box plots depict the median (line), upper and lower quartiles (box) ±1.5× interquartile range (whiskers); outliers not shown.

#### Processing of ORFtag screens

First, iPCR reads from sorted and background (nonselected, input) samples were trimmed using Trim galore (v.0.6.0) with default parameters to remove Illumina adapters. Then, trimmed reads were aligned to the mm10 version of the mouse genome using Bowtie2 (ref. ^21^) (v.2.3.4.2) with default parameters (for paired-end sequenced samples, only first mate reads were considered), before removal of duplicated and low mapping quality reads (mapq ≤ 30) using samtools (v.1.9)^22^. Mapped insertions were assigned to the closest downstream exon junction—with a maximum distance of 200 kb—based on GENCODE annotations of the mouse genome (vM25). Finally, insertion counts were aggregated per gene. Of note, only exons from protein-coding transcripts were considered, except for the first exon of each transcript, which does not contain splicing acceptor sites. Consequently, intronless genes—for which none of the isoforms contain a spliced intron—were not considered.

Background replicates showed reproducible gene counts (PCC ≥ 0.84) and therefore were merged, and genes with at least one insertion were considered as putatively tagged. Finally, genes showing significantly more insertions in sorted samples compared to merged background samples were identified using one-tailed Fisher’s exact test (alternative = ‘greater’) on merged biological replicates. Of note, only genes with at least three unique insertions in sorted samples were considered. Obtained *P* values were corrected for multiple testing using the FDR method and genes showing an FDR < 0.001 and a log_2_ odds ratio ≥1 were classified as hits.

On visual inspection, we noticed that certain gene loci (for example, Morc1) had strand-symmetrical insertion patterns in the sorted samples, similar to background and/or input. These patterns could indicate true positives (if two hits are closely located in the genome with reverse orientations), but could also represent false positives. Therefore, we implemented a flag to suggest that these hits are treated with caution (‘Enriched for reversed integrations’ in the comment column, Supplementary Table 1). To do this, we flipped the strand of integrations before assigning them to the closest downstream, nonfirst exon. Only genes showing a significant enrichment (FDR < 0.001 and log_2_ odds ratio ≥1) for such reversed integrations in sorted versus input samples were flagged. The flags were rare: in the activator screen, four out of 139 hits were flagged, all known activators (Ldb1, Ss18, Taf4b, Pprc1). Similarly, only 11 out of 207 hits in the repressor screen were flagged, including eight known repressors (Trim28, Zbtb45, Zfp472, Zfp85, Zfp74, Zfp568, Zfp493, Zfp799) and three less-well-characterized proteins (3300002I08Rik, Gm10130, Gm10324). Finally, the PTGR screen had ten out of 77 hits flagged (Trim28, Morc1, Sfi1, Pou5f1, Virma, Esrp1, Zbtb45, Zic3, Esp38, Gm5485).

#### Frame-specific ORFtag screens

For each mouse exon assigned to a protein-coding gene, the phase of overlapping coding sequence (CDS) was retrieved from the GENCODE annotation (vM25). For exons starting upstream of the first CDS of the transcript, their phase was corrected to reflect the number of nucleotides separating the ATG of the ORFtag cassette and the endogenous ATG of the spliced transcript (for example, an exon starting two nucleotides upstream of the first ATG of a transcript would have a corrected phase of 2).

To assess whether different ORFtag cassettes show a bias toward in-frame exons, the three frame-specific activator ORFtag screens (frames 1–3) were analyzed in parallel and assigned to the closest downstream exon, as described in the previous section. Finally, we compared the frame of the cassette (frames 1–3) to the phase of the assigned exons (phases 2, 1, 0, respectively) to assess whether spliced transcripts would encode in-frame products.

#### Processing of ORFtag transcripts and prediction of in-frame transcripts

First, ORFtag RNA sequencing (RNA-seq) reads were separated depending on the frame of the cassette, using regular expression matching anchored at the start of reads’ sequence (frame 1: ^[NACGT]{6}CCACGACGGAGACTACAAGGATCATGATATTGATTACAAAGACGATGACGATAAGCAG, frame 2: ^[NACGT]{6}CCACGACGGAGACTACAAGGATCATGATATT--GATTACAAAGACGATGACGATAAGGCAG, frame 3: ^[NACGT]{6}CCACGACGGAGA--CTACAAGGATCATGATATTGATTACAAAGACGATGACGATAAGGCCAG), allowing for one mismatch. Then, the constant sequences were trimmed before mapping the remaining part of the reads—corresponding to the first spliced exon—to the mm10 version of the mouse genome. Finally, the frame of each read was compared to the phase of overlapping exons, which were corrected to reflect the number of nucleotides separating the ATG of the ORFtag cassette and the first CDS downstream of the splice-acceptor site. Finally, the frame of the cassette (frames 1–3) was compared to the corrected phase of the assigned exons (phases 2, 1, 0, respectively) to assess whether spliced transcripts would encode in-frame products.

#### Protein–protein interaction networks

For each functional assay, STRING protein–protein interactions between hits were retrieved using the STRINGdb R package (v.2.10.0, database v.11.0). Finally, only the hits showing at least one protein–protein interaction with another hit with a combined score ≥900 were considered. This threshold was set to ensure easy visual inspection of resulting plots.

#### CDS length bias

To assess whether ORFtag is biased toward short ORFs, we stratified intronic protein-coding genes based on their shortest CDS length (<2.5, 2.5–5 and longer than 5 kb). Then, we compared how tagged genes (with at least one insertion in background samples) and hits (union from the three screens) were distributed between these groups, using all intronic protein-coding genes as a reference. For example, to compute the normalized ratio of tagged genes for the <2.5 kb group, we used the following formula: normalized ratio = ((tagged genes with CDS < 2.5 kb)/(total tagged genes))/((intronic protein genes with CDS < 2.5 kb)/(total intronic protein-coding genes)). To allow side-by-side comparison, we also considered ORFs from the human ORFeome that Alerasool and colleagues were able to transfect and detect^2^.

#### Gene expression bias

To assess whether transcriptionally inactive mouse genes could be assayed using ORFtag, we used publicly available data from the same mES cell line (GSE99971)^23^. For each intronic protein-coding gene, mean transcripts per kilobase per million (TPM) was computed across three RNA-seq replicates (only protein-coding genes were considered). Genes with a mean TPM of 0 were classified as inactive and active genes were further stratified into quartiles. Then, we compared how tagged genes (with at least one insertion in background samples) and hits (union from the three screens) were distributed between these groups, using all intronic protein-coding genes as a reference. For example, to compute the normalized ratio of tagged genes for the inactive group, we used the following formula: normalized ratio = ((tagged genes with TPM = 0)/(total tagged genes))/((intronic protein genes with TPM = 0)/(total intronic protein-coding genes)).

#### Enrichment analysis of publicly available gene sets

To assess whether ORFtag or ORFeome hits were enriched for genes with expected functions, we collected publicly available lists of human transcription factor genes^24^, human genes containing activation or repressive domains (ref. ^25^; only genes containing sufficient (‘S’ or ‘N and S’) and high confidence (‘H’) domains were considered), human genes that were hits in the ORFeome activator screen^2^, human genes containing RNA-binding domains (RBPbase^26^; only the genes identified in at least two different cell lines were used) and human fusion oncoproteins (COSMIC database v.97, ref. ^27^). ORFtag hits were first assigned to their human orthologs using MGI^28^ homology data. For each functional assay, we assessed whether relevant categories were enriched among the hits using one-tailed Fisher’s exact test (alternative = ‘greater’), and the total number of intronic protein-coding genes as background.

#### GO terms and protein domains enrichment

Biological process, molecular process and cellular component GO terms were obtained from the org.Mm.eg.db (v.3.15.0) R package. Protein domains were retrieved from the EnsDb.Mmusculus.v.79 R package (v.2.99.0). For each functional assay, GO terms and protein domains that were over-represented among hits were identified using one-tailed Fisher’s exact test (alternative = ‘greater’), using all intronic protein-coding genes as background. Obtained *P* values were corrected for multiple testing using the FDR method and features with an FDR < 0.05 were considered as significantly enriched. Of note, small categories containing fewer than five genes in total and categories with fewer than three matching hits were not considered. Finally, the top 8–10 enriched GO terms and proteins domains were plotted for each functional assay.

#### Protein family enrichment

To identify protein families enriched among mouse intronless genes (for which none of the isoforms contain a spliced intron), annotations were retrieved from the EnsDb.Mmusculus.v79 R package (v.2.99.0). Enriched protein families were identified using one-tailed Fisher’s exact test (alternative = ‘greater’), and the total number of protein-coding genes as background. Obtained *P* values were corrected for multiple testing using the FDR method, and the protein families with an FDR < 0.05 were plotted.

#### Analysis of first exons and predicted CDS fraction of protein products

For the analysis of first exons in mouse genes, first exons containing a predicted CDS were classified as either short (≤20 amino acids) or long (>20 amino acids). Then, manually curated Pfam-A domains from UCSC^29^ were used to discriminate first exon CDSs containing a know protein domain (that is, coding for at least 10% of a full annotated protein domain from the Pfam database) or not. For each integration, we compared the start of assigned exons (above) to start of transcripts’ CDS to assess whether the tagged product would contain the full-length protein or a truncated version. In the last case, we distinguished short truncations (<10% of the coding sequence) from major ones (>10%).

#### Gene annotation for PRO-seq analysis

To obtain a nonredundant set of genes for quantification of PRO-seq signals, we collected all coding and long noncoding transcripts from Ensembl v.100 for the mm10 version of the mouse genome, excluding transcripts shorter than 300 bp. When several transcript isoforms shared the same annotated TSS, only the longest isoform was retained. Next, TSS positions were corrected using FANTOM5 (ref. ^30^) CAGE TSS clusters: for each unique annotated TSS, we identified the strongest CAGE TSS within a 1 kb window centered on the annotated TSS, excluding the coding sequence. Finally, for each CAGE TSS, only the full length of the nearest transcript was used to count overlapping reads (next section).

#### PRO-seq analysis

PRO-seq libraries were sequenced in paired-end mode with 36 bp read lengths. To eliminate PCR duplicates, an 8 bp long unique molecular identifier (UMI) was incorporated at the 5′ end of the reads during the sample processing. Before mapping, the UMI was separated, and the Illumina adapters were trimmed using cutadapt v.1.18. Only reads with a length greater than 10 bp were then mapped using Bowtie v.1.2.2 (ref. ^31^), initially to the mm10 version of the mouse genome. The mapping allowed for up to two mismatches and reported only the best alignment (-m 1–best–strata) for each read. To ensure the counting of unique nascent RNA molecules, reads that mapped to the same genomic location were collapsed based on their UMIs, allowing for up to one mismatch. To create the PRO-seq coverage signal with the exact positions of RNA pol II molecules, only the first nucleotide of each read (that is, the 3′ end of nascent transcripts) was considered and the strand swapped to match the transcription direction. A nonredundant CAGE-corrected gene set was used to count the number of UMI-collapsed 1 nt-long mapped PRO-seq reads that overlap them (‘Gene annotation for PRO-seq analysis’ section). Differential analysis was performed using DESeq2 (ref. ^32^) (v.1.22.2) and significantly up- or downregulated genes were selected using FDR < 0.05, log_2_ fold change ≥1 threshold. To ensure accurate quantification of transcriptional changes and to potentially detect global effects, spike-in-based normalization was used. The normalization scaling factor was calculated based on the relative abundance of remaining reads that mapped to the spike-in genome (dm3) in combined replicates for each condition and supplied to DESeq2, with all replicates of the same condition receiving the same scaling factor. These scaling factors were also applied to normalize the PRO-seq coverage of combined replicates per condition, allowing for visualization in the genome browser.

#### Cut&Run analysis

Single-end 50 bp long reads were mapped to the mm10 genome using Bowtie v.0.12.9, allowing up to three mismatches and only uniquely mapping reads were retained. Afterward, peaks were called for each individual replicate, as well as for the combined replicates against their respective input, using Macs2 v.2.1.2.1, with following settings: -f BEDPE -g mm -B–nomodel–extsize 300–SPMR. The Macs2 generated BedGraph files that contain normalized coverage were converted into BigWig using bedGraphToBigWig. Given the high correlation between two replicates (PCC of 0.613 at a common set of peaks), only the merged sample was used for assigning bound genes if the peak was localized within ±500 bp around the gene TSSs.

### Reporting summary

Further information on research design is available in the Nature Portfolio Reporting Summary linked to this article.

## Online content

Any methods, additional references, Nature Portfolio reporting summaries, source data, extended data, supplementary information, acknowledgements, peer review information; details of author contributions and competing interests; and statements of data and code availability are available at 10.1038/s41592-024-02339-x.

## Supplementary information

Supplementary Information Supplementary Figs. 1 and 2.

Reporting Summary

Supplementary Table 1 Identification of activator, repressor and PTGR hits. For each gene locus, raw counts, odd ratio (log_2_) and the associated FDR are shown for the three different screens. The ‘hit’ column specifies whether a locus was considered as a hit (TRUE) or not (FALSE). The ‘comment’ column contains flagged gene loci that are enriched for reversed integrations (Methods).

## Source data

Source Data Fig. 2 Unprocessed western blots for Fig. 2e.

Source Data Fig. 3 Unprocessed western blots for Fig. 3d.

## Data Availability

The raw sequencing data generated in this study are available from the Gene Expression Omnibus (GEO) (https://www.ncbi.nlm.nih.gov/geo/) under accession number GSE225972. These data were aligned to the mouse reference genome (mm10) available at https://www.ncbi.nlm.nih.gov/datasets/genome/GCF_000001635.20/. The annotations for the mouse genome were sourced from GENCODE (v.M25, https://www.gencodegenes.org/mouse/release_M25.html) and Ensembl (v.100, https://nov2020.archive.ensembl.org/Mus_musculus/Info/Annotation). Previously published datasets referenced and used in this study are detailed in the Methods section and are available as follows: GEO accession number GSE99971 (RNA-seq)^23^; list of transcription factor genes^24^; list of genes containing activation or repressive domains^2^; list of hits in the ORFeome activator screen^25^; list of genes containing RNA-binding domains^26^; list of fusion oncoproteins^27^; human–mouse orthologs^28^ and manually curated Pfam-A domains. No restrictions on data availability apply. Source data are provided with this paper.

## Code availability

All custom scripts that were generated for this study were made publicly available at https://github.com/vloubiere/ORFtag_2024.
