# Supplementary material for: Proteome-scale tagging and functional screening in mammalian cells by ORFtag
Source: Nat Methods. 2024 Jul 5;21(9):1668–73. doi: 10.1038/s41592-024-02339-x (PMC11399080; doi:10.1038/s41592-024-02339-x)
Supplement: Supplementary file 1 — Supplementary Figs. 1 and 2. [file 41592_2024_2339_MOESM1_ESM.pdf]

nature methods

Supplementary information https://doi.org/10.1038/s41592-024-02339-x

Proteome-scale tagging and functional screening in mammalian cells by ORFtag

In the format provided by the authors and unedited

Supplementary Figure 1: Figure exemplifying the gating strategy

Activator ORFtag reporter cell line

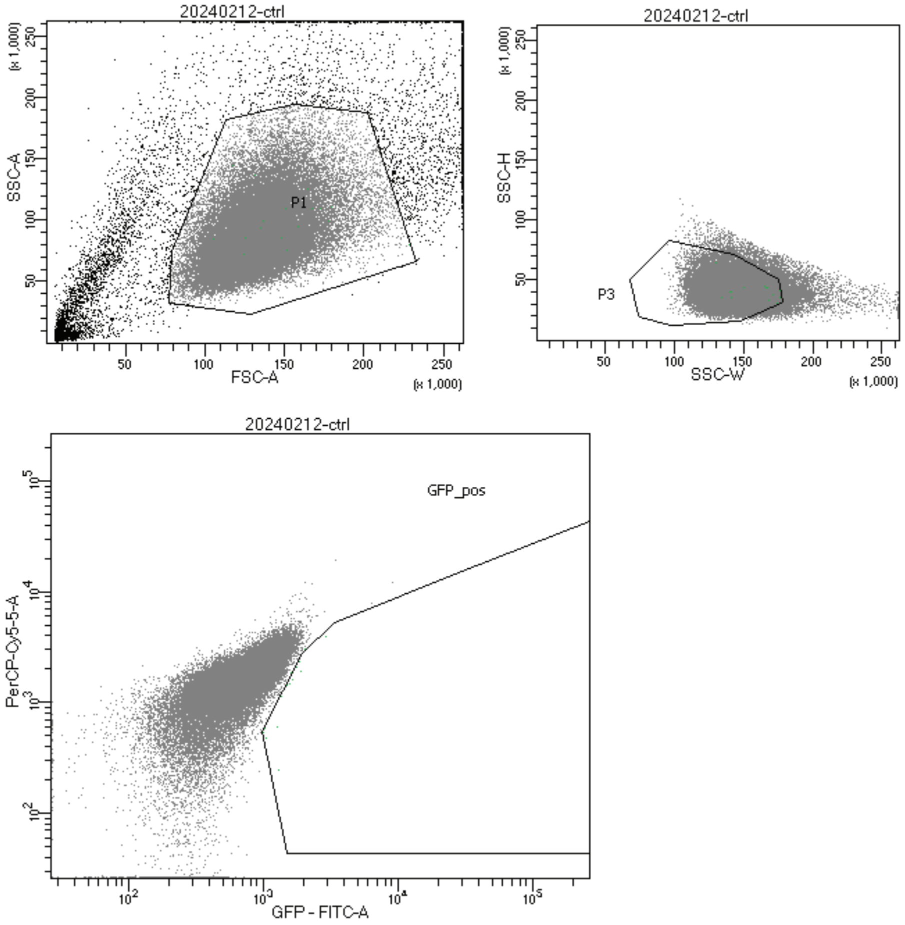

Activator ORFtag reporter cell line + ORFtag virus

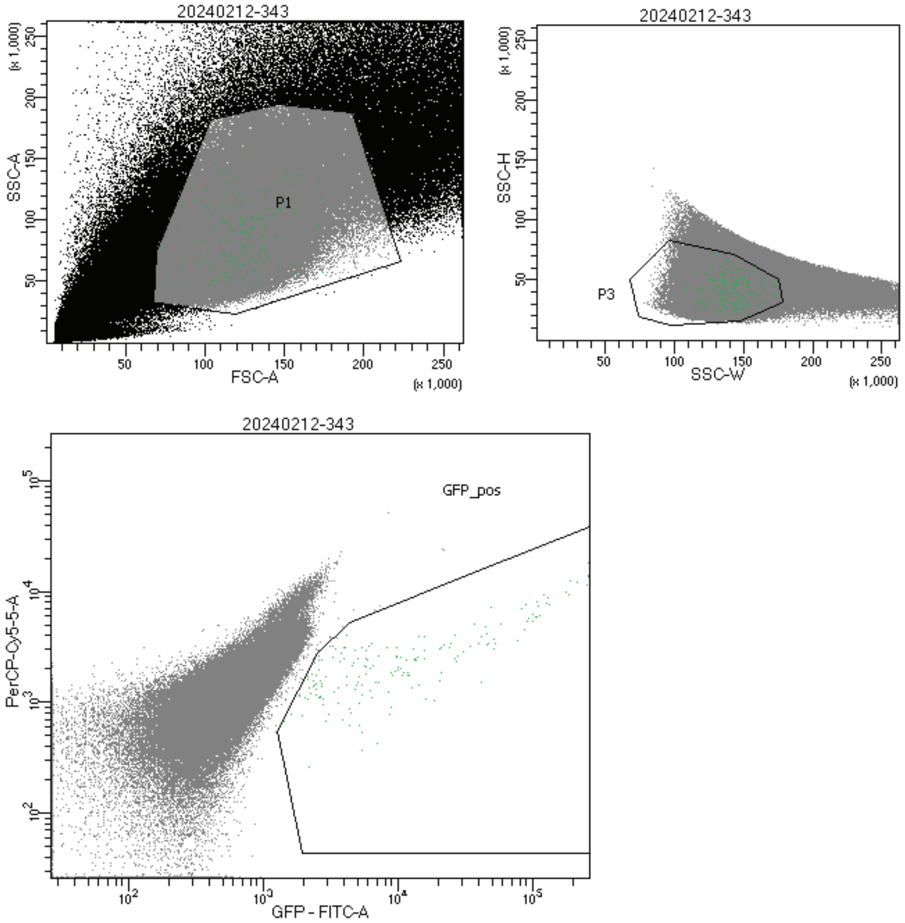

Supplementary Figure 2: List of primers used in the study

name	sequence	description
KRAB–F	ATCATGATATTGATTACAAGGATGACGATGACAAGGCGATGGATGCTAAGTCACTAACTGC	cloning TetR–KRAB using Gibson cloning
KRAB–R	GTAGCTCCGCTTCCGGCGGCGGGCTCTTCTCCCTTCTC	cloning TetR–KRAB using Gibson cloning
Jdp2–F	ATTGATTACAAGGATGACGATGACAAGGCAATGATGCCTGGGCAGATCCC	cloning TetR–Jdp2 using Gibson cloning
Jdp2–R	GCTGAAGTTAGTAGCTCCGCTTCCGGCGGCCTTCTTGTCCAGCTGCTCC	cloning TetR–Jdp2 using Gibson cloning
Etv6–F	ATTGATTACAAGGATGACGATGACAAGGCAATGTCTGAGACTCCTGCTCAG	cloning TetR–Etv6 using Gibson cloning
Etv6–R	GCTGAAGTTAGTAGCTCCGCTTCCGGCGGCTTCCCGGGTCTCTTCCTTTAC	cloning TetR–Etv6 using Gibson cloning
N4bp1–F	ATTGATTACAAGGATGACGATGACAAGGCAATGGCGGCCCGGGTGGTG	cloning TetR–N4bp1 using Gibson cloning
N4bp1–R	GCTGAAGTTAGTAGCTCCGCTTCCGGCGGCGTCCAACACCAGGGCCGAC	cloning TetR–N4bp1 using Gibson cloning
Rbpj–F	ATTGATTACAAGGATGACGATGACAAGGCAATGCCCTCCGGTTTTCCTCA	cloning TetR–Rbpj using Gibson cloning
Rbpj–R	GCTGAAGTTAGTAGCTCCGCTTCCGGCGGCGGACACCACGGTTGCTGTG	cloning TetR–Rbpj using Gibson cloning
Msantd3–F	ATTGATTACAAGGATGACGATGACAAGGCAATGCAAAACAACGAAATCATTAAACC	cloning TetR–Msantd3 using Gibson cloning
Msantd3–R	GCTGAAGTTAGTAGCTCCGCTTCCGGCGGCGGGTGAATTGGGAAAGGGC	cloning TetR–Msantd3 using Gibson cloning
Trim8–F	ATTGATTACAAGGATGACGATGACAAGGCAATGGCGGAGAATTGGAAGAACTG	cloning TetR–Trim8 using Gibson cloning
Trim8–R	GCTGAAGTTAGTAGCTCCGCTTCCGGCGGCGCTTGTCACATAGTGTTTGGTGG	cloning TetR–Trim8 using Gibson cloning
Rnf220–F	ATTGATTACAAGGATGACGATGACAAGGCAATGGACTTACACCGGGCAGC	cloning TetR–Rnf220 using Gibson cloning
Rnf220–R	GCTGAAGTTAGTAGCTCCGCTTCCGGCGGCTAAGTAGATCCTCCGCAGGTC	cloning TetR–Rnf220 using Gibson cloning
Tox4–F	ATTGATTACAAGGATGACGATGACAAGGCAATGGAGTTTCCCGGAGGAAATG	cloning TetR–Tox4 using Gibson cloning
Tox4–R	GCTGAAGTTAGTAGCTCCGCTTCCGGCGGCCTTCACAAACACCACAGAGTTTGG	cloning TetR–Tox4 using Gibson cloning
DsRed–F	GGGGACAAGTTTGTACAAAAAAGCAGGCTTCATGGCCTCCTCCGAGGACGTCAT	cloning TetR–DsRed using Gateway cloning
DsRed–R	GGGGACCACTTTGTACAAGAAAGCTGGGTCCAGGAACAGGTGGTGGCGGCCCT	cloning TetR–DsRed using Gateway cloning
VPR–F	GGGGACAAGTTTGTACAAAAAAGCAGGCTTCGACGCATTGGACGATTTTGATC	cloning TetR–VPR using Gateway cloning
VPR–R	GGGGACCACTTTGTACAAGAAAGCTGGGTCAAACAGAGATGTGTCGAAGATG	cloning TetR–VPR using Gateway cloning
Lmo4–F	GGGGACAAGTTTGTACAAAAAAGCAGGCTTCATGGTGAATCCGGGCAGCAGCTCGC	cloning TetR–Lmo4 using Gateway cloning
Lmo4–R	GGGGACCACTTTGTACAAGAAAGCTGGGTCGCAGACCTTCTGGTCTGGCAGTAGT	cloning TetR–Lmo4 using Gateway cloning
Agfg1–F	GGGGACAAGTTTGTACAAAAAAGCAGGCTTCATGGCGGCCAGCGCGAAGCGGAAGC	cloning TetR–Agfg1 using Gateway cloning
Agfg1–R	GGGGACCACTTTGTACAAGAAAGCTGGGTCTAAGAAAGGGTTGGTGGATGAGCTT	cloning TetR–Agfg1 using Gateway cloning
Tsx–F	GGGGACAAGTTTGTACAAAAAAGCAGGCTTCATGTCTGAAAAGCAAAGCCCCAAGA	cloning TetR–Tsx using Gateway cloning
Tsx–R	GGGGACCACTTTGTACAAGAAAGCTGGGTCATCAGTTGGGTTCATGGCACCATCT	cloning TetR–Tsx using Gateway cloning
Cited1–F	GGGGACAAGTTTGTACAAAAAAGCAGGCTTCATGCCAACTATGTCGAGGCCTGCAC	cloning TetR–Cited1 using Gateway cloning
Cited1–R	GGGGACCACTTTGTACAAGAAAGCTGGGTCGCAGCCAGAGGGAAAATCTGCAGTG	cloning TetR–Cited1 using Gateway cloning
Zc3h7b–F	GGGGACAAGTTTGTACAAAAAAGCAGGCTTCATGGAGAGGCAGAAACGGAAGGCGG	cloning TetR–Zc3h7b using Gateway cloning
Zc3h7b–R	GGGGACCACTTTGTACAAGAAAGCTGGGTCCTCCCCAGTGGTGGTGGTGGCAGCA	cloning TetR–Zc3h7b using Gateway cloning
1700102P08Rik–F	GGGGACAAGTTTGTACAAAAAAGCAGGCTTCATGTCTGAAAAACTACGAAGATGCA	cloning TetR–1700102P08Rik using Gateway cloning
1700102P08Rik–R	GGGGACCACTTTGTACAAGAAAGCTGGGTCCTCTATTTGGTATTTAAAAGAAGTC	cloning TetR–1700102P08Rik using Gateway cloning
Bicral–F	GGGGACAAGTTTGTACAAAAAAGCAGGCTTCATGGATGATGACGATGACTCCTGTC	cloning TetR–Bicral using Gateway cloning
Bicral–R	GGGGACCACTTTGTACAAGAAAGCTGGGTCACACTCTAGAATACTATTTACAGCT	cloning TetR–Bicral using Gateway cloning
Pxn–F	GGGGACAAGTTTGTACAAAAAAGCAGGCTTCATGGACGACCTCGATGCCCTGCTGG	cloning TetR–Pxn using Gateway cloning
Pxn–R	GGGGACCACTTTGTACAAGAAAGCTGGGTCGCAGAAGAGCTTCACGAAGCAGCTC	cloning TetR–Pxn using Gateway cloning
BFP_F	TACAAAGACGATGACGATAAGCAGGAATTCAGCGAGCTGATTAAGGAGAAC	cloning lambdaN–BFP using Gibson cloning
BFP_R	AGTTAGTAGCTCCGCTTCCTCTAGAATTAAGCTTGTGCCCCAGTTTG	cloning lambdaN–BFP using Gibson cloning
Tnrc6b–SD_F	TACAAAGACGATGACGATAAGCAGGAATTCCAAGGAGGAAAGAAACAAATGAAG	cloning lambdaN–Tnrc6b–SD using Gibson cloning
Tnrc6b–SD_R	AGTTAGTAGCTCCGCTTCCTCTAGAGATTGAATCCGACCCTCCTC	cloning lambdaN–Tnrc6b–SD using Gibson cloning
Ncoa5_F	AGACGATGACGATAAGCAGGAATTCAATACGGCTCCATCAAGACC	cloning lambdaN–Ncoa5 using Gibson cloning
Ncoa5_R	AGTTAGTAGCTCCGCTTCCTCTAGAGTAATGCCTCTGGTAAGATCCC	cloning lambdaN–Ncoa5 using Gibson cloning
Maco1_F	TACAAAGACGATGACGATAAGCAGGAATTCAAGCGGCGGAACGCCGAC	cloning lambdaN–Maco1 using Gibson cloning
Maco1_R	AGTTAGTAGCTCCGCTTCCTCTAGACTTCTTCAAGGGCTGGTAGACAGAG	cloning lambdaN–Maco1 using Gibson cloning
Trim13_F	TACAAAGACGATGACGATAAGCAGGAATTCGAGCTGCTTGAAGAAGAC	cloning lambdaN–Trim13 using Gibson cloning
Trim13_R	AGTTAGTAGCTCCGCTTCCTCTAGATAATAGTTTGTATTTGCATATAAACTCTG	cloning lambdaN–Trim13 using Gibson cloning
Trim26_F	TACAAAGACGATGACGATAAGCAGGAATTCGCAGTGTCAGCCCCCTTG	cloning lambdaN–Trim26 using Gibson cloning
Trim26_R	AGTTAGTAGCTCCGCTTCCTCTAGAGGGTCTCAGCAGAAGGCGTG	cloning lambdaN–Trim26 using Gibson cloning
Trim25_F	TACAAAGACGATGACGATAAGCAGGAATTCGCGGAGCTGAATCCTCTG	cloning lambdaN–Trim25 using Gibson cloning
Trim25_R	AGTTAGTAGCTCCGCTTCCTCTAGATTTGGAGCAGATAGAGAGGG	cloning lambdaN–Trim25 using Gibson cloning
Mib2_F	TACAAAGACGATGACGATAAGCAGGAATTCGACCTGGACCCCCATGCAG	cloning lambdaN–Mib2 using Gibson cloning
Mib2_R	AGTTAGTAGCTCCGCTTCCTCTAGACACGAAGATCTGAATGCGGTC	cloning lambdaN–Mib2 using Gibson cloning
Trim71dE1_F	TACAAAGACGATGACGATAAGCAGGAATTCGTCCTGCATCTCTACTGTGAC	cloning lambdaN–Trim71dE1 using Gibson cloning
Trim71dE1_R	AGTTAGTAGCTCCGCTTCCTCTAGAGAAGATGAGGATTCGATTGTTGC	cloning lambdaN–Trim71dE1 using Gibson cloning
Tnip1_F	AGACGATGACGATAAGCAGGAATTCGAAGGGAGAGGACCCTACG	cloning lambdaN–Tnip1 using Gibson cloning
Tnip1_R	AGTTAGTAGCTCCGCTTCCTCTAGACTGGGGCCCATCACAGTC	cloning lambdaN–Tnip1 using Gibson cloning
Chr15–RMCE–F	CGCCCAGAAGCTAATCTGAAGGGCTGC	genotyping primer for Chr15–RMCE locus
Chr15–RMCE–R	ATGAGTCATGCTATTCTCTAATCATG	genotyping primer for Chr15–RMCE locus
ORFtag–RNAseq–RT	GTGACTGGAGTTCAGACGTGTGCTCTTCCGATCTNNNNNN	ORFtag–RNAseq reverse transcription primer
ORFtag–RNAseq–nested–F	TATGTGGCCTGGAGAAACAGCTA	ORFtag–RNAseq nested PCR primer
ORFtag–RNAseq–nested–R	GTGACTGGAGTTCAGACGTGTGCTCTTCCGATCT	ORFtag–RNAseq nested PCR primer
ORFtag–RNAseq–final–F	CACGACGCTCTTCCGATCTNNNNNNCCACGACGGAGACTACAAGG	ORFtag–RNAseq final PCR primer
ORFtag–cDNA–seq	CCTGGCAATCGAGATGCTGGACAG	ORFtag clone cDNA sequencing
Zfp574–genotyping–F	TGATGGACAGGGCTTTGGGAACCAG	genotyping AID–tagged Zpf574 clones
Zfp574–genotyping–R	TGCGGTGCCATCATCTTCAGGTGTA	genotyping AID–tagged Zpf574 clones
ORFtag–nested–F	TGCAGGACCGGACGTGACTGGAGTTC*A	ORFtag nested PCR primer
ORFtag–nested–R	TGCAGGACGATGAGCAGAGCCAGAACC*A	ORFtag nested PCR primer
ORFtag–iPCR–F	AATGATACGGCGACCACCGAGATCTACACGAGCCAGAACCAGAAGGAACTTGA*C	ORFtag inverse PCR primer
ORFtag–iPCR–R	CAAGCAGAAGACGGCATACGAGAT–[xxxxxx]–GTGACTGGAGTTCAGACGTGTGCTCTTCCGATCT	ORFtag inverse PCR primer with a barcode
ORFtag–sequencing–1	GAGTGATTGACTACCCGTCAGCGGGGGTCTTTCA	ORFtag Illumina sequencing primer 1
ORFtag–sequencing–2	TGAGTGATTGACTACCCACGACGGGGGTCTTTCA	ORFtag Illumina sequencing primer 2
