# Supplementary material for: Proteome-scale tagging and functional screening in mammalian cells by ORFtag
Source: Nat Methods. 2024 Jul 5;21(9):1668–73. doi: 10.1038/s41592-024-02339-x (PMC11399080; doi:10.1038/s41592-024-02339-x)
Supplement: Supplementary file 2 — Reporting Summary [file 41592_2024_2339_MOESM2_ESM.pdf]

nature portfolio

Corresponding author(s): Julius Brennecke, Ulrich Elling, Alexander Stark, Stefan L. Ameres

Last updated by author(s): May 7, 2024

Reporting Summary

Nature Portfolio wishes to improve the reproducibility of the work that we publish. This form provides structure for consistency and transparency in reporting. For further information on Nature Portfolio policies, see our Editorial Policies and the Editorial Policy Checklist.

Statistics

For all statistical analyses, confirm that the following items are present in the figure legend, table legend, main text, or Methods section.

n/a	Confirmed	
☐	☒	The exact sample size (*n*) for each experimental group/condition, given as a discrete number and unit of measurement
☐	☒	A statement on whether measurements were taken from distinct samples or whether the same sample was measured repeatedly
☐	☒	The statistical test(s) used AND whether they are one- or two-sided<br>*Only common tests should be described solely by name; describe more complex techniques in the Methods section.*
☒	☐	A description of all covariates tested
☐	☒	A description of any assumptions or corrections, such as tests of normality and adjustment for multiple comparisons
☐	☒	A full description of the statistical parameters including central tendency (e.g. means) or other basic estimates (e.g. regression coefficient) AND variation (e.g. standard deviation) or associated estimates of uncertainty (e.g. confidence intervals)
☐	☒	For null hypothesis testing, the test statistic (e.g. *F*, *t*, *r*) with confidence intervals, effect sizes, degrees of freedom and *P* value noted<br>*Give P values as exact values whenever suitable.*
☒	☐	For Bayesian analysis, information on the choice of priors and Markov chain Monte Carlo settings
☒	☐	For hierarchical and complex designs, identification of the appropriate level for tests and full reporting of outcomes
☐	☒	Estimates of effect sizes (e.g. Cohen's *d*, Pearson's *r*), indicating how they were calculated

Our web collection on statistics for biologists contains articles on many of the points above.

Software and code

Policy information about availability of computer code

Data collection	Western Blot images were taken using ChemiDocTM Imaging Systems, Bio-Rad. Flow cytometry experiments were run on iQue Screener PLUS, Intellicyt and FACS LSR Fortessa, BD. Next generation sequencing was NGS was performed on an Illumina NextSeq550 or llumina HiSeq 2500 sequencer.
Data analysis	Biorad ImageLab (v5.1.1), Trim galore (v0.6.0), GenomicRanges (v1.50.1), Bowtie2 (v2.3.4.2), bowtie (v0.12.9 & v1.2.2), samtools (v1.9), Macs2 (v2.1.2.1), cutadapt (v1.18), IGV (v2.16.0), FlowJo v10.10, R (v4.2.0)<br>R packages: data.table (v1.14.6), STRINGdb R package (v2.10.0, database v11.0), org.Mm.eg.db (v3.15.0), EnsDb.Mmusculus.v79 (v2.99.0), DESeq2 (v1.22.2), flowCore (v2.12.2)<br>Genome annotation: GENCODE mouse (vM25), Ensembl mouse (v100/mm10)<br><br>All custom scripts that were generated for this study were made publicly available at https://github.com/vloubiere/ORFtag_2024.

For manuscripts utilizing custom algorithms or software that are central to the research but not yet described in published literature, software must be made available to editors and reviewers. We strongly encourage code deposition in a community repository (e.g. GitHub). See the Nature Portfolio guidelines for submitting code & software for further information.

Data

Policy information about availability of data

All manuscripts must include a data availability statement. This statement should provide the following information, where applicable:

- Accession codes, unique identifiers, or web links for publicly available datasets
- A description of any restrictions on data availability
- For clinical datasets or third party data, please ensure that the statement adheres to our policy

The raw sequencing data generated in this study are available from GEO (https://www.ncbi.nlm.nih.gov/geo/) under accession number GSE225972. These data were aligned to the mouse reference genome (mm10) available at https://www.ncbi.nlm.nih.gov/datasets/genome/GCF_000001635.20/. The annotations for the mouse genome were sourced from GENCODE (vM25, https://www.gencodegenes.org/mouse/release_M25.html) and Ensembl (v100, https://nov2020.archive.ensembl.org/Mus_musculus/Info/Annotation). Previously published datasets referenced and used in this study are detailed in the methods section and are available as follows: GEO accession number GSE99971 (RNA-seq), Ref.23 (list of transcription factor genes), Ref.24 (list of genes containing activation or repressive domains), Ref.2 (list of hits in the ORFeome activator screen), Ref.25 (list of genens containing RNA-biding domains), Ref.26 (list of fusion oncoproteins), Ref.27 (human – mouse orthologs), Ref.28 (manually-curated Pfam-A domains). No restrictions on data availability apply.

Human research participants

Policy information about studies involving human research participants and Sex and Gender in Research.

Reporting on sex and gender	N/A
Population characteristics	N/A
Recruitment	N/A
Ethics oversight	N/A

Note that full information on the approval of the study protocol must also be provided in the manuscript.

Field-specific reporting

Please select the one below that is the best fit for your research. If you are not sure, read the appropriate sections before making your selection.

☒ Life sciences ☐ Behavioural & social sciences ☐ Ecological, evolutionary & environmental sciences

For a reference copy of the document with all sections, see nature.com/documents/nr-reporting-summary-flat.pdf

Life sciences study design

All studies must disclose on these points even when the disclosure is negative.

Sample size	Sample size was defined in compliance with the gold standards of the field, such that relevant statistical parameters would get stabilized. Recruitment assays were performed by measuring 25,000 cells for each validation, except for N4bp1 (n=5,766) and Trim8 (n=3,775) - given the validation setup, cells represent independent events. ORFtag screens were performed in 2 biological replicates. We show that integrations are consistent across not only replicates, but also 6 independent experiments with PCC≥0.84. Additional validations of the ORFtag strategy, namely frame-specific ORFtag screens and ORFtag-RNA-seq were performed once. Cut&Run was performed in two biological replicates following ENCODE's standards for transcription factor occupancy (https://www.encodeproject.org/chip-seq/transcription-factor-encode4/#standards). PRO-seq was performed in two biological replicates following ENCODE's standards for transcriptomics (https://www.encodeproject.org/data-standards/encode4-bulk-rna/#standards).
Data exclusions	For transcriptome differential expression analysis, only the genes with at least 0.05 tagcount normalized tags per per kb per million, that separates expressed gene, were retained for differential analysis, complying with good practices.
Replication	The reproducibility of our experimental findings has been confirmed by performing independent biological replicates. PRO-seq: 2 biological replicates; ORFtag screens: 2 biological replicates; Cut&Run: 2 biological replicates; Recruitment assays: 1 experiment with random virus integration of tested constructs - each measured cell is an independent observation; Competition assay: 2 biological replicates; Validation of Zfp574 depletion: 2 biological replicates. Additional validations of the ORFtag strategy, namely frame-specific ORFtag screens and ORFtag-RNA-seq were performed once. All replication attempts were successful.
Randomization	Not relevant because the samples were not grouped.
Blinding	Blinding was not used in our experiments as it is not relevant - the need for transparency in identifying control samples for accurate data analysis precludes the use of blinding. However, we have maintained an unbiased approach in our data analysis to avoid confirmation bias and subjective interpretations of data.

Reporting for specific materials, systems and methods

We require information from authors about some types of materials, experimental systems and methods used in many studies. Here, indicate whether each material, system or method listed is relevant to your study. If you are not sure if a list item applies to your research, read the appropriate section before selecting a response.

Materials & experimental systems

n/a	Involved in the study
☐	☒ Antibodies
☐	☒ Eukaryotic cell lines
☒	☐ Palaeontology and archaeology
☒	☐ Animals and other organisms
☒	☐ Clinical data
☒	☐ Dual use research of concern

Methods

n/a	Involved in the study
☒	☐ ChIP-seq
☐	☒ Flow cytometry
☒	☐ MRI-based neuroimaging

Antibodies

Antibodies used: mouse α-Flag M2 (Sigma Aldrich F3165, 1:10,000), mouse α-V5-tag (Thermo Fisher R960-25, 1:1,000), rabbit α-β-tubulin (Abcam, ab6046, 1:10,000), HRP-α-Mouse (Cell Signaling, 7076, 1:10,000), HRP-α-Rabbit (Cell Signaling, 7074, 1:10,000)

Validation: All antibodies are commercial, widely used, and were validated by the manufacturer:
mouse α-Flag M2 (Sigma Aldrich F3165): https://www.sigmaaldrich.com/AT/en/product/sigma/f3165; Monoclonal ANTI-FLAG® M2 detects 2 ng of FLAG-BAP™ fusion protein on a dot blot using chemiluminescent detection. Monoclonal ANTI-FLAG® M2 detects a single band of protein on a Western blot from an E.coli crude cell lysate.

mouse α-V5-tag (Thermo Fisher R960-25): This antibody is functionally tested against 20 ng of an E. coli expressed fusion protein containing a V5 epitope using a chemiluminescent substrate at a 1 minute exposure. This antibody has also been tested in Western blot against 25 ng of recombinant Positope™ protein. The Positope™ control protein is a 53 kDa recombinant protein that contains seven epitope tags, including His (C-term), HisG, c-myc, and V5. Low background was observed using chemiluminescent or alkaline phosphatase reagents for detection. Using chemiluminescence as the detection method, no cross-reactivity has been observed in bacterial lysates. In mammalian lysates, a few cross-reactive proteins have been observed upon overexposure of blots.

rabbit α-β-tubulin (Abcam ab6046): Abcam says that "We have tested this species and application combination and it works. It is covered by our product promise.". This antibody detects a single clean band at 50kD representing beta Tubulin. This band is significantly reduced by using peptide blocking. https://www.abcam.com/en-at/products/primary-antibodies/beta-tubulin-antibody-loading-control-ab6046#

HRP-α-Mouse (Cell Signaling, 7076): Affinity purified horse anti-mouse IgG (heavy and light chain) antibody is conjugated to horseradish peroxidase(HRP) for chemiluminescent detection. This product is thoroughly validated with CST primary antibodies and will work optimally with the CST western immunoblotting protocol, ensuring accurate and reproducible results.

HRP-α-Rabbit (Cell Signaling, 7074): Designed for use with rabbit polyclonal and monoclonal antibodies, this affinity purified goat anti-rabbit IgG (heavy and light chain) antibody is conjugated to horseradish peroxidase(HRP) for chemiluminescent detection. This product is thoroughly validated with CST primary antibodies and will work optimally with the CST western immunoblotting protocol, ensuring accurate and reproducible results.

Eukaryotic cell lines

Policy information about cell lines and Sex and Gender in Research

Cell line source(s): AN3-12 mouse embryonic stem cells is a cell line created in the Elling lab (Elling 2017, 10.1038/nature24027) and was obtained from IMBA Haplobank. PlatE cells were purchased from Cell Biolabs. LentiX cells were obtained from Clontech. Drosophila S2 cells were purchased from Thermo Fisher (#R69007).

Authentication: Visual inspection was used to confirm the morphology of cell lines

Mycoplasma contamination: All cell lines are negative for mycoplasma.

Commonly misidentified lines (See ICLAC register): No commonly misidentified cell lines were used in this study.

Flow Cytometry

Plots

Confirm that:

☒ The axis labels state the marker and fluorochrome used (e.g. CD4-FITC).

☒ The axis scales are clearly visible. Include numbers along axes only for bottom left plot of group (a 'group' is an analysis of identical markers).

☒ All plots are contour plots with outliers or pseudocolor plots.

☒ A numerical value for number of cells or percentage (with statistics) is provided.

Methodology

Sample preparation	For flow cytometric analysis, cells were trypsinized and resuspended in medium.
Instrument	Intellicyt iQue Screener PLUS, BD FACS LSRFortessa
Software	R, v4.2.0 and package flowCore, v2.12.2, FlowJo v10.10
Cell population abundance	Cell numbers are included within the manuscript: the sample size was 25,000 cells for each validation, except for N4bp1 (n=5,766) and Trim8 (n=3,775).
Gating strategy	FSC/SSC gating was used to exclude dead cells and cell fragments and to analyze single cells.

☒ Tick this box to confirm that a figure exemplifying the gating strategy is provided in the Supplementary Information.
