# Supplementary figures and images for: Proteome-scale tagging and functional screening in mammalian cells by ORFtag
Source: Nat Methods. 2024 Jul 5;21(9):1668–73. doi: 10.1038/s41592-024-02339-x (PMC11399080; doi:10.1038/s41592-024-02339-x)

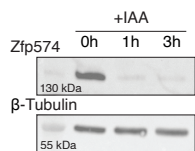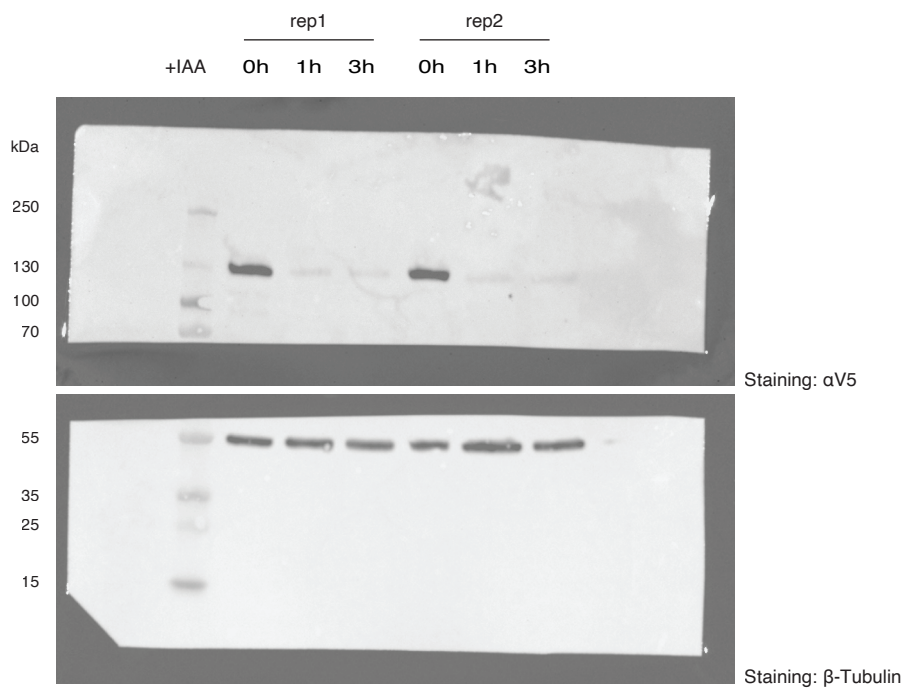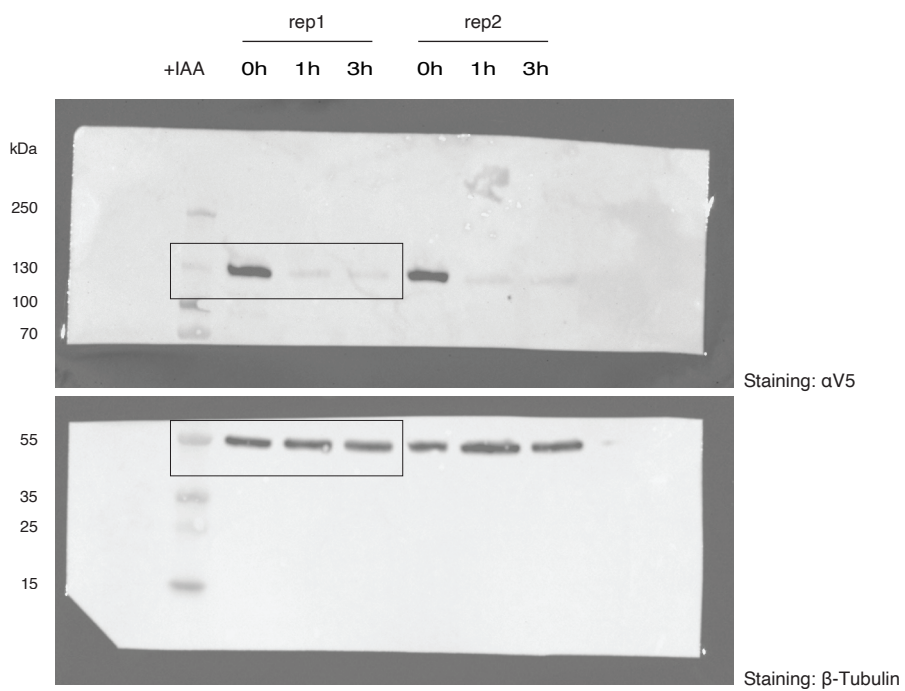

Supplement: Supplementary file 4 — Unprocessed western blots for Fig. 2e. [file 41592_2024_2339_MOESM4_ESM.pdf]

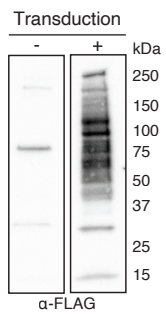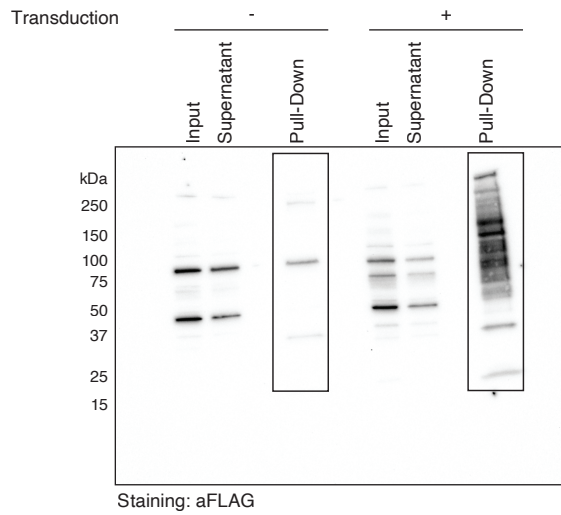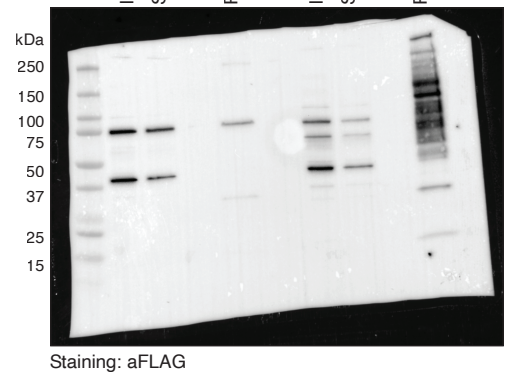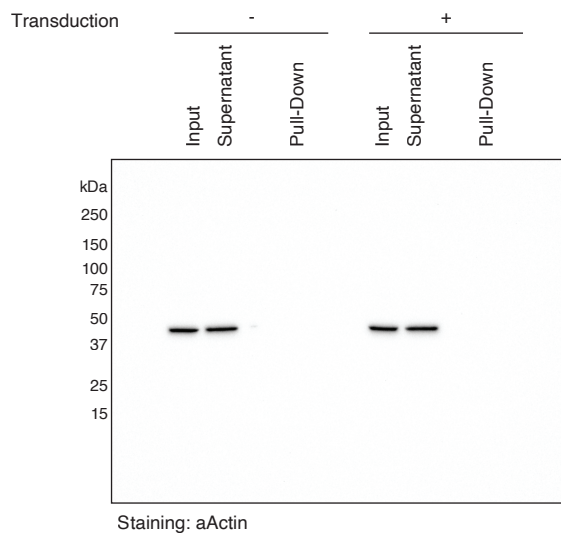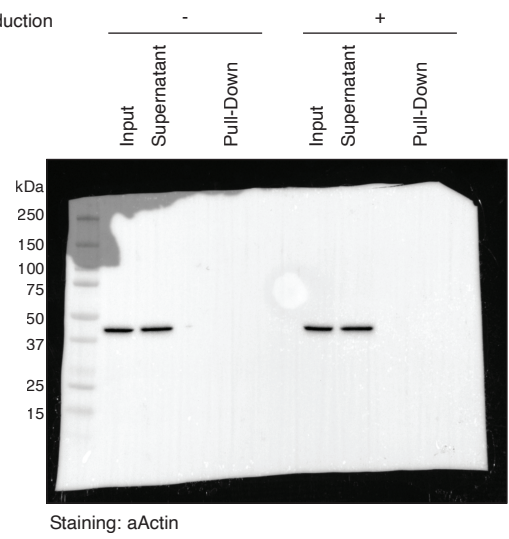

Supplement: Supplementary file 5 — Unprocessed western blots for Fig. 3d. [file 41592_2024_2339_MOESM5_ESM.pdf]
